# Biofilm aggregates and the host airway-microbial interface

**DOI:** 10.3389/fcimb.2022.969326

**Published:** 2022-08-23

**Authors:** Luanne Hall-Stoodley, Karen S. McCoy

**Affiliations:** ^1^ Department of Microbial Infection and Immunity, The Ohio State University College of Medicine, Columbus, OH, United States; ^2^ Division of Pulmonary Medicine, Nationwide Children’s Hospital, Columbus, OH, United States

**Keywords:** biofilm, bacteria, human respiratory tract, human, respiratory infection, cystic fibrosis, otitis media

## Abstract

Biofilms are multicellular microbial aggregates that can be associated with host mucosal epithelia in the airway, gut, and genitourinary tract. The host environment plays a critical role in the establishment of these microbial communities in both health and disease. These host mucosal microenvironments however are distinct histologically, functionally, and regarding nutrient availability. This review discusses the specific mucosal epithelial microenvironments lining the airway, focusing on: i) biofilms in the human respiratory tract and the unique airway microenvironments that make it exquisitely suited to defend against infection, and ii) how airway pathophysiology and dysfunctional barrier/clearance mechanisms due to genetic mutations, damage, and inflammation contribute to biofilm infections. The host cellular responses to infection that contribute to resolution or exacerbation, and insights about evaluating and therapeutically targeting airway-associated biofilm infections are briefly discussed. Since so many studies have focused on *Pseudomonas aeruginosa* in the context of cystic fibrosis (CF) or on *Haemophilus influenzae* in the context of upper and lower respiratory diseases, these bacteria are used as examples. However, there are notable differences in diseased airway microenvironments and the unique pathophysiology specific to the bacterial pathogens themselves.

## The human airway epithelium

**Figure 1:**
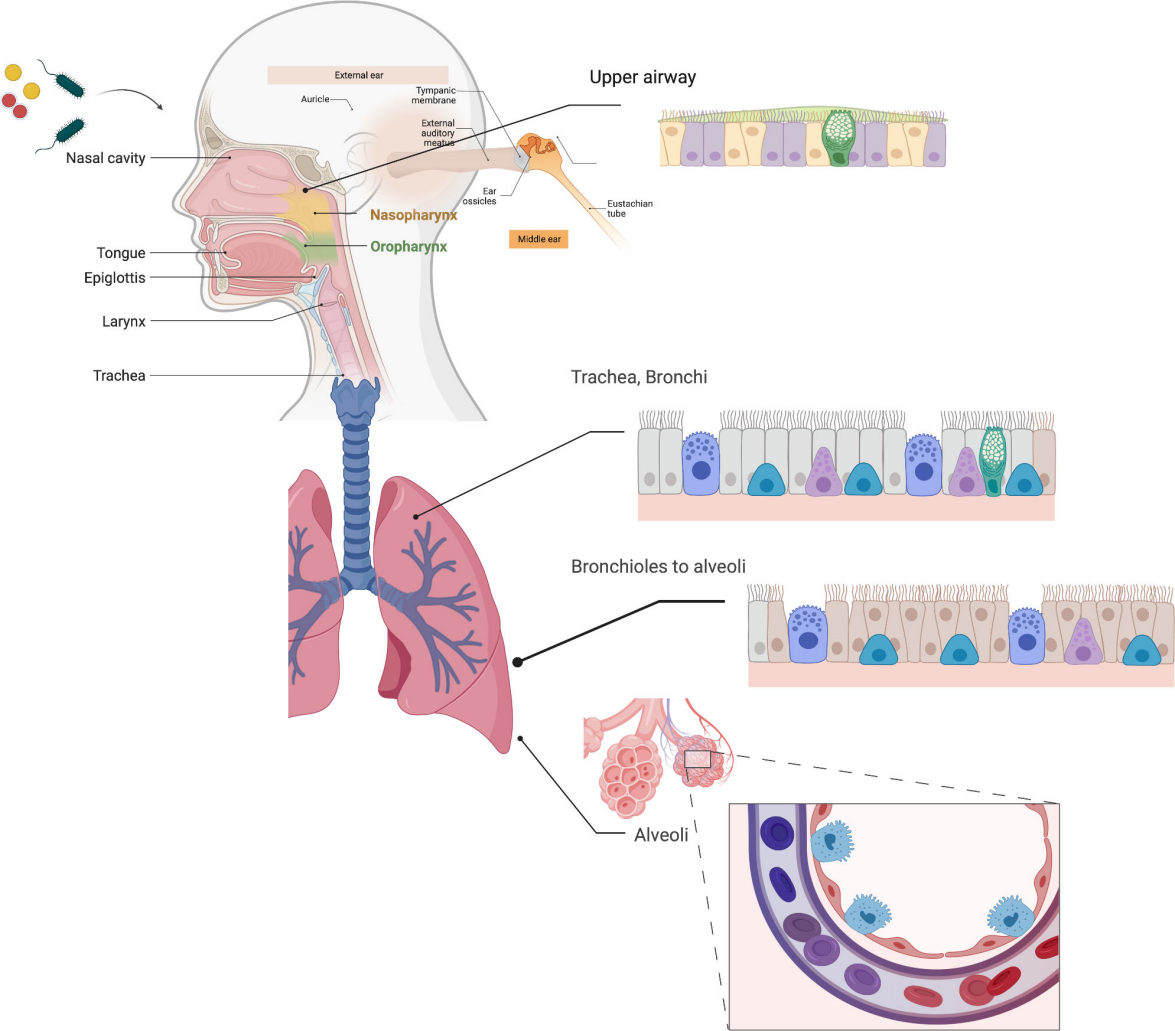
Respiratory Epithelia. The respiratory epithelia include the nasal epithelium, nasopharynx, middle ear and eustachian tubes, oropharynx, and the pulmonary epithelium from the trachea to alveoli. Much of the respiratory epithelium is pseudostratified columnar epithelium, and unique to the airway, and quite distinct from other mucosal epithelia found in in the genitourinary and intestinal epithelium. Ciliated cells (purple/salmon) and goblet cells (GC), which secrete mucins (green) are found throughout the respiratory epithelium, while type 1 (red) and type II (cyan) alveolar epithelial cells are found in the distal airways. Created with BioRender.com.

Breathing exposes the airway to microbes and microbial byproducts including many environmental pathogens as well as various opportunists from commensal oral and nasopharyngeal microbiota. The airway mucosal epithelium consists of structurally heterogeneous cell types, including stratified non-cornified squamous epithelium, which covers the pharyngeal and palatine surfaces in the oropharynx, ciliated pseudostratified columnar epithelium, simple columnar and cuboidal epithelium in bronchioles, and ending in the squamous alveolar epithelium, the exquisitely thin cellular interface that promotes gas exchange. The ciliated pseudostratified columnar epithelium or “respiratory epithelium” comprises most of the respiratory tract and is found in nasal passages, the middle ear, the trachea, and bronchi (**Figure 1**). The airway transports liters of air through the upper respiratory tract and conducting airways to the alveoli. Epithelial cells vitally function as a protective barrier against pathogens and irritants to avert infection, inflammation, and tissue injury. This barrier function is helped by mucus secreting goblet cells, which act in concert with ciliated cells to promote ciliary clearance by entrapping bacteria and particulates within the airway and expelling them *via* the mucociliary escalator. To maintain homeostasis, the airway must deliver oxygen to the systemic circulation despite the risk of microbes and irritants breaching the diverse physical, chemical, and host innate defense systems that have evolved to prevent their entry.

Notably, the respiratory tract is mechanically dynamic (Hall-Stoodley et al., 2006b; Gloag et al., 2018; Gloag et al., 2021). Substantial shear forces occur in the larger conducting airways (Levitsky, 1982), whereas the cells lining the alveoli in the terminal airways respond to micromechanical forces and shear from expansion and contraction that changes with lung tidal volume, i.e., bulging of the epithelium at low volumes and surface tensions (contraction) and stretching of the epithelium at higher tidal volumes (expansion) (Bachofen and Wilson, 1997). In the respiratory epithelium, mucociliary clearance is driven largely by cilia, however as the airways branch into smaller conducting pathways, the reduced airflow and shear leads to mucus effectively gliding along the epithelial surface (Boucher, 2019). In contrast, increased airflow and higher shear forces in the larger airways can lead to the breakdown of the cohesive forces of mucus to better facilitate sputum clearance by cough (Boucher, 2019). The airway physiology is therefore multifaceted with specific conditions such as oxygen tension, shear stress, and specialized cellular microenvironments, which act as a key interface for host-pathogen interactions.

## Commonalities in the respiratory epithelial microenvironment

Mucus and cilia are key components comprising the mucociliary escalator in conducting airways. Ciliated cells are the dominant cell type of the human respiratory epithelium, which distinguishes primate airways from murine species where secretory cells predominate (Whitsett and Alenghat, 2015). Specifically, the cellular composition and organization of the intrapulmonary epithelium in mice is restricted to the most distal portions of human conducting airways, while the mouse trachea is much more comparable to of the human lung (Rock et al., 2010). These differences may account for a shortcoming of many mouse models to recapitulate chronic bacterial respiratory infections with biofilm-forming organisms without infecting preformed aggregated bacteria in an artificial matrix (Thomsen et al., 2021). Attached to the basal lamina, basal cells are present below the surface epithelium along the conducting airways. Basal cells act as progenitors of both ciliated cells and secretory (goblet cells) and play a critical role in homeostasis and regenerating the airway epithelium following infection or injury (Whitsett, 2018). Basal cells and the regulatory cascades responsible for repair and regeneration in the conducting airway epithelium are also distinct (Reynolds et al., 2008; Ghosh et al., 2011; Ghosh et al., 2013). The pseudostratified epithelium of the tracheobronchial airways contains multiple progenitor cell populations that are discrete compared to the subpopulations in simple epithelium of bronchioles (Hong et al., 2004; Ghosh et al., 2011; Whitsett, 2018) and these tracheobronchial subpopulations, which function in homeostasis, are heterogeneous *in vivo*. Club cells constitute approximately a fifth of cells found in terminal and respiratory bronchioles and are a primary secretory cell in the human small airway epithelium (Dean and Snelgrove, 2018). Club cells produce secretory surfactants (surfactant proteins A, B, and D) and proteins that contribute to the airway epithelial lining fluid (Harkema et al., 2018). As club cells also can serve as progenitor cells for ciliated and secretory epithelial cells, they too play a role in airway cellular homeostasis. These specialized cells make up the cellular components of mucociliary defense in the respiratory airway. Goblet, basal, and club cells secrete fluids and host-defense proteins into the airway lumen and ciliated cells propel the mucus so that it can be eliminated in the respiratory tract.

In the conducting airways several host defense molecules are involved in the protection of airway homeostasis. These include defensins, lysozyme, lactoferrin, (Singh et al., 2002; Rogan et al., 2004), LL-37 (the sole cathelicidin found in human airways) (Overhage et al., 2008; Sibila et al., 2019), and short-palate lung and nasal epithelial clone 1 (SPLUNC1), which is a multifunctional innate defense protein. Surfactant proteins SP-A and SP-D, which also play an important role in innate immunity, are also produced in the lower airway by type II alveolar epithelial cells (Ferguson and Schlesinger, 2000; Crowther and Schlesinger, 2006; Hall-Stoodley et al., 2006b). Surfactant proteins can facilitate bacterial aggregation and act as opsonins that may promote phagoctytosis. SPLUNC1 for example found in the conducting airways and upper respiratory tract has been shown to affect biofilm formation by *P. aeruginosa* (Liu et al., 2013). Studies in SPLUNC1 KO mice indicate that the absence of this molecule led to suppression of several epithelial secretory proteins and antimicrobial molecules that affected mucociliary clearance, decreased the innate immune response, and increased biofilm formation by *P. aeruginosa*. At physiologically relevant concentrations, purified recombinant human SPLUNC was shown to enhance the surfactant function of airway secretions and anti-biofilm activity against *P. aeruginosa* (Gakhar et al., 2010). While knock down of SPLUNC1 failed to impact survival of NTHi in the chinchilla model of otitis media, it was essential for maintenance of middle ear pressure and efficient mucociliary clearance (McGillivary and Bakaletz, 2010). Nitric oxide (NO) is a signaling molecule that is produced by epithelial cells and other cells in the respiratory tract that has been shown to induce biofilm dispersal with multiple bacteria (Allan et al., 2016; Römling, 2019; Allan et al., 2017; Collins et al., 2017; Walker et al., 2014; Walker et al., 2017; Howlin et al., 2017). However, NO has complex roles in bacteria, including the production of oxygen by denitrification in *P. aeruginosa* at the onset of anaerobiosis, which might affect its adaptation under anoxic conditions (Lichtenberg et al., 2021). Airway defense molecules thus are regulated by exposure to pathogens and their byproducts (such as LPS), by cytokines from the airway tissue microenvironment, and by underlying lung disease (Walker et al., 2014; Whitsett and Alenghat, 2015; Walker et al., 2017; Tecle et al., 2010; Kim et al., 2018).

Secretory immunoglobulin A (S-IgA) produced by plasma (immunoglobulin secreting cells) lining the airway epithelium is the major immunoglobulin component of human mucosal surfaces and contributes to preventing pathogen adherence and promoting clearance in the airway. While less studied in the respiratory tract compared to the gut mucosa, IgA appears to be important in homeostasis and controlling inflammation (Sánchez Montalvo et al., 2022). IgA is produced as a dimer and transcytosed from the basal epithelial side to the apical surface by the polymeric immunoglobulin receptor (pIgR) to form S-IgA, which functions to neutralize many viral pathogens and some bacteria and with airway mucus play a role in agglutinating microorganisms (Gohy et al., 2014; de Fays et al., 2022). Ciliated, goblet cells, and club cells express pIgR and importantly mucosal S-Ig-A is altered in both upper and lower airway diseases (de Fays et al., 2022).

**Figure 2:**
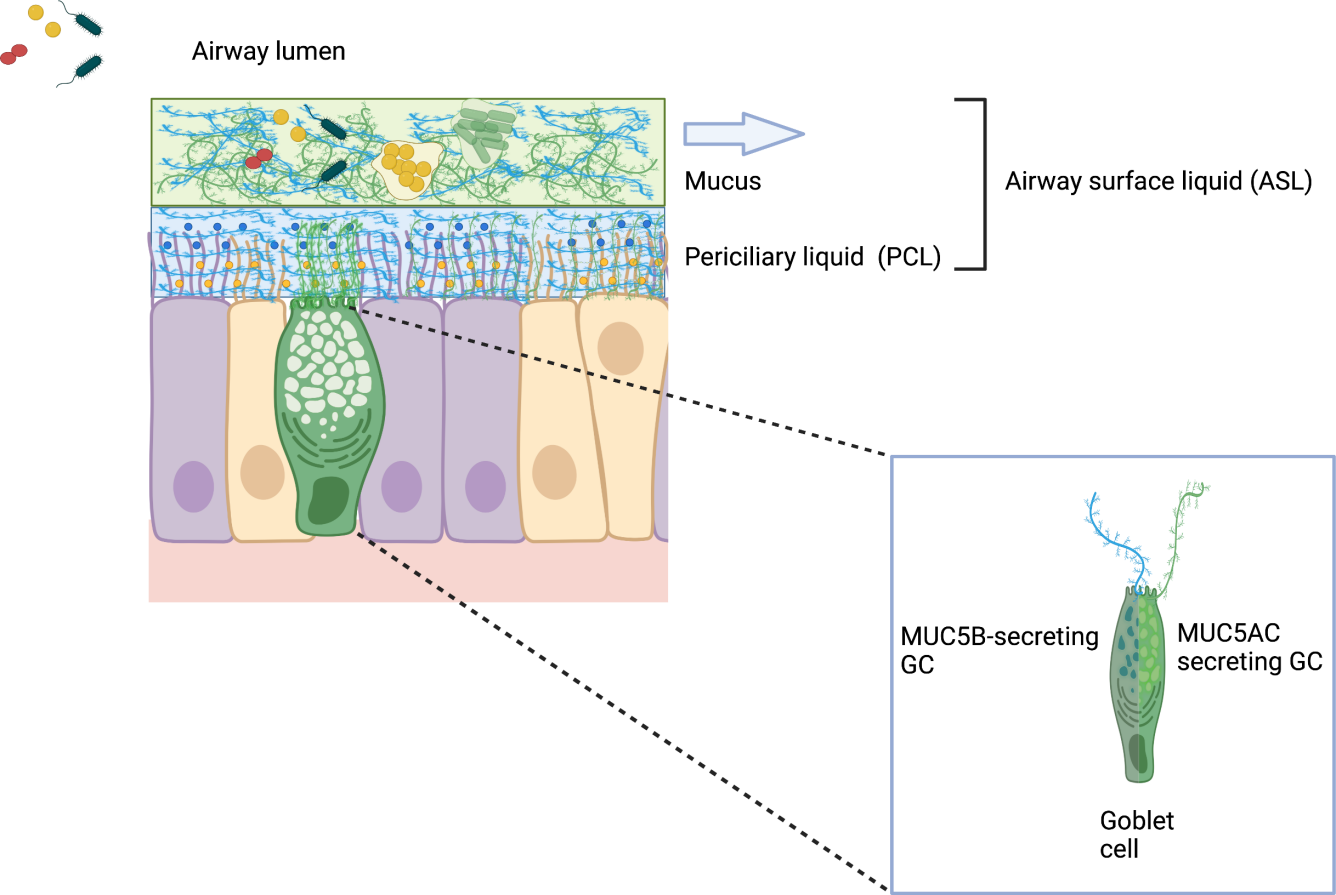
Gel-on-Brush model of the respiratory epithelial mucus clearance system. The Gel-on-Brush representation best describes the cell biology of two layers depicting the ASL and the biophysical interactions between these layers that control mucus clearance. This model is consistent with data showing that the ASL is comprised of two hydrogel layers that limit penetration of particles and bacteria into the periciliary liquid (PCL) overlying the epithelial cells. PCL contains membrane-spanning mucin glycoproteins and large mucopolysaccharides that may be tethered to cilia and the epithelial surface or secreted by goblet cells (inset). Mucin subunits form larger macromonomer chains in the mucus layer. Tethered mucins (not depicted in this schematic) form an extracellular brush resulting in a mesh-like hydrogel that prevents large macromolecules (both MUC5AC/B mucins) in the mucus layer and inhaled bacteria encountering the airway surface from penetrating the PCL. The higher concentration of membrane-tethered macromolecules in the extracellular brush layer produces inter-molecular repulsion within the PCL to ensure that the PCL is not compressed by the osmotically dynamic mucus layer. Notably, the stability of the PCL forms the distinct mucus layer necessary for effective mucus clearance (arrow), while dehydration destabilizes the two-layer system and compromises clearance. (Wagner et al., 2018; Hill et al., 2022). Created with BioRender.com.

Mucus is a vital component of the respiratory epithelium. Studies using differentiated human bronchial epithelial (HBE) cell cultures suggest the airway mucus layer is suspended above a periciliary layer (PCL) by mucins and mucopolysaccharides that span the airway surface epithelial cells. This “gel-on-brush” model indicates that mucus forms a gel-like layer that can entrap particles yet allows small molecules to infiltrate the PCL (Button et al., 2012; Hill et al., 2022). This model indicates the PCL is not a liquid stratum, but rather functions as a macromolecular gel mesh, conducive for ciliary beating, that can also prevent bacteria and large virus particles from efficiently accessing the epithelial cell surface (**Figure 2**). Importantly, this periciliary hydrogel consists of mucins, MUC1, MUC4, and MUC16, which are tethered to epithelial cell surface and cilia. Both hydrogel strata generate an osmotic microenvironment defined by an osmotic modulus, which regulates hydration by varying the concentration of macromolecules. In health, the higher differential osmotic pressure of the PCL enables it to sufficiently bathe the airway epithelial surface and promote efficient ciliary beating and transport of the overlying mucus hydrogel compartment (Boucher, 2019). In disease however, high osmotic moduli either due to the increased concentration of mucins, which can occur in COPD, or decreased hydration, which occurs in CF, lead to compression of the PCL brush (Morrison et al., 2022). These changes in differential osmotic pressure can functionally compromise underlying cilia in their ability to promote effective mucociliary clearance.

Hydrated glycosylated protein mucins can be divided into three types; cell-associated mucins, which are anchored to the epithelial cell surface, non-polymerizing secreted mucins, and gel-forming mucins (Whitsett, 2018). The airway mucus hydrogel above the PCL primarily contains the respiratory mucins MUC5AC and MUC5B. These relatively large glycopeptides are found in mucus associated with the middle ear, nasal, and lung mucosal epithelium forming the polymeric structure of the hydrogel-like mesh (Boucher, 2019). MUC5B is thought to be constitutively produced and the dominant mucin by concentration, whereas MUC5AC is induced by external stresses such as irritants and infection (Collin et al., 2021). Interestingly, while agglutination and entrapping microbes may appear to be passive, native mucins play a recently described active role in a robust barrier function of healthy mucus. This gel lattice can actively reduce bacterial adherence, disrupt bacterial aggregation, bind to host defense proteins, and suppress virulence in several pathogens (Wheeler, 2019) and inhibits large numbers of pathogens from reaching the epithelial cell surface and breaching the mucus airway barrier. Studies with the gel-forming constituents of mucus and the opportunistic pathogens *P. aeruginosa* and *Candida albicans* have shown that mucus profoundly affected motility, intracellular signaling, and suppressed the expression of virulence traits including surface attachment, biofilm formation, and horizontal gene transfer (Wheeler et al., 2019). In this regard mucins in the healthy airways function much more than simply a passive physical barrier that eradicates pathogens by the mucociliary escalator.

Studies that have examined the components of mucus and their biophysical or mechanical properties are leading to a further important understanding of how a disease microenvironment can be modulated (Wagner et al., 2018). Rheological studies of the mechanical properties of mucus have produced further insights into the protective barrier function. Inasmuch as mucus has properties of both a liquid and a gel, mechanically it is defined as a viscoelastic solid (Lai et al., 2009). Under conditions of low shear, mucus behaves like an elastic solid that can regain its shape over time, whereas under conditions of high shear, mucus behaves like a viscous liquid that can irreversibly deform. Thus, mucus is elastic at physiological shear rates in the upper airways, which preserves the structure of the mucus lattice network, and nasal mucus is more viscoelastic than mucus from the large airways, underlining the importance of the anatomical microenvironment (Lai et al., 2009). These biophysical properties are important in effecting the release and expulsion of mucus by mucociliary clearance or cough. For an excellent comprehensive overview of human airway mucus see (Hill et al., 2022).

## Biofilm aggregates – attached or not – display antibiotic tolerance

Interestingly, both facets of the airway host-microbial interface employ multicellular strategies for survival and persistence. Biofilms are multicellular aggregated bacterial communities that are phenotypically distinct from single-celled (planktonic) bacteria. Bacterial aggregates are typically embedded in a matrix of extracellular polymeric substances (EPS) consisting of protein, carbohydrate, extracellular DNA (eDNA), and lipids (Limoli et al., 2015; Irie et al., 2012; Koo et al., 2017). Although eDNA can be self-produced by bacteria (Whitchurch, 2002; Allesen-Holm et al., 2006; Hall-Stoodley et al., 2008; Jurcisek et al., 2017), eDNA is also present in inflamed airways from host neutrophils and can be incorporated into the *P. aeruginosa* extracellular biofilm matrix (Moreau-Marquis et al., 2008; Walker, 2005; Jennings et al., 2021). In a microscopic study that included a CF lung tissue-section, host-derived eDNA was primarily found surrounding *P. aeruginosa* biofilms but not part of the bacterial biofilm matrix *per se* suggesting that *in vivo* host-derived eDNA may form a “secondary” matrix, which further impedes antibiotic penetration (Alhede et al., 2020a).

**Figure 3:**
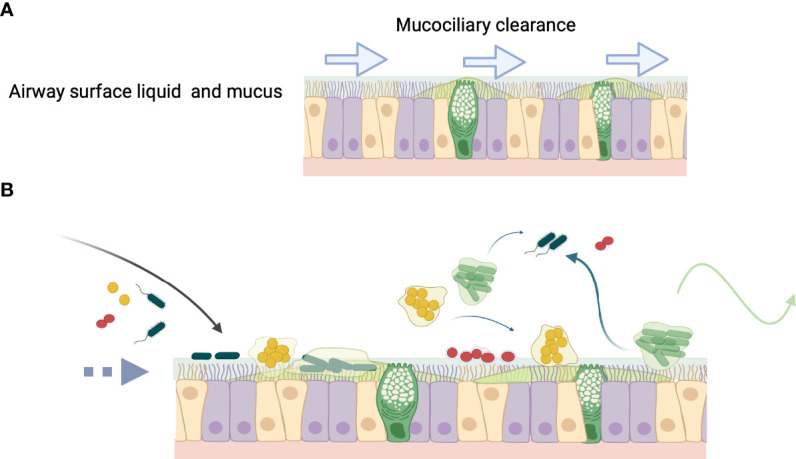
The fate of bacteria in the airway. **(A)** Normally airway surface liquid (ASL) (blue) and mucus (green) produced by secretory cells, bathe the epithelial surface and are swept along by ciliated cells, which contribute to mucociliary clearance (MCC) denoted by arrows. **(B)** In inflammatory lung diseases or during infection that diminishes MCC (broken arrow), bacteria enter the airway (black arrow) and may colonize mucus, which becomes a niche for the development of biofilm aggregates. Aggregated bacteria can clonally proliferate on the epithelial surface as in chronic otitis media, or form aggregates that are present in the bronchial epithelial secretions comprising sputum, as in cystic fibrosis lung infection. Biofilm aggregates may result from changes in ASL osmotic concentration and destabilization of the hydrogel layers (see **Figure 2**). Mucin glycans can play an important role in governing microbial interactions, such as in receptor binding sites for bacterial adhesion, biofilm formation, providing nutrient sources, and environmental signaling. (Wagner et al., 2018; Hill et al., 2022). Aggregates or single bacteria can propagate to other areas within the respiratory epithelium (blue wispy arrows), for example from the upper to the lower respiratory tract or can be cleared from the airway by cough (green arrow).

Biofilm-associated infections are common and can be found in airway diseases where they can resist host immune responses and antibiotic therapies. Although often cited for the ability of biofilms to persist *in vivo*, demonstrating *pathogen-specific* biofilm phenotypes *ex vivo* in clinical samples requires techniques that identify specific pathogens (Singh et al., 2000; Hall-Stoodley et al., 2006a; Bjarnsholt et al., 2009; Hall-Stoodley et al., 2012; Jennings et al., 2021). Numerous examples of bacterial aggregates adherent to underlying epithelial cells have been shown on *ex vivo* biopsies from upper airway infections, e.g., chronic otitis media and chronic sinusitis (Sanclement et al., 2005; Hall-Stoodley et al., 2006a; Sanderson et al., 2006; Foreman et al., 2009; Thornton et al., 2011; Thornton et al., 2013). In lower airway infections, such as CF pneumonia, studies with nonciliated small airway epithelial cells, such as A549 cells, indicate that *P. aeruginosa* and *H. influenzae* can adhere to epithelial cells (Chi et al., 1991; Häußler et al., 2003; Starner et al., 2006; Byrd et al., 2010). Bacterial aggregates are also found *ex vivo* suspended in the inspissated mucus layer, rather than directly adherent to the epithelial cell surface (**Figure 3**) (Worlitzsch et al., 2002; Bjarnsholt et al., 2009). Studies with differentiated human bronchial epithelial cells suggest that mucus helps to protect the underlying epithelial cell surface, where bacteria within the mucus hydrogel layer are subjected to biophysical properties that govern efficient mucus clearance (Gloag et al., 2018; Wheeler et al., 2019; Gloag et al., 2021) (discussed further below). Importantly, biofilm aggregates can occur independently of adherence to epithelial cells.

Regardless of whether they are adherent or suspended, the higher order multicellular organization of microbial aggregates can contain functionally diverse bacterial phenotypes including dormant or metabolically inactive cells that enhance the ability of bacteria to withstand diverse unfavorable environments, such as oxidative stress and antibiotic treatment (Boles et al., 2004; Hall-Stoodley et al., 2004; Boles and Singh, 2008; Koo et al., 2017; Crabbé et al., 2019; Stewart, 2015). Aggregates can exhibit greater fitness due to better access to nutrients than single cells (Kragh et al., 2016). Crucially, aggregates can tolerate concentrations of antibiotic that exceed the minimum inhibitory or bactericidal concentrations required to eradicate bacteria (Singh et al., 2000; Fux et al., 2004; Alhede et al., 2011; Allan et al., 2016; Kragh et al., 2018; Clary, 2020). While the EPS matrix may not inhibit the penetration of antibiotics into the biofilm altogether, it can slow the rate of penetration enough to allow bacteria to upregulate and express genes that mediate resistance such as efflux pumps or β-lactamases (Jefferson et al., 2005; Stewart, 1996). For example, calculations of β-lactam antibiotic penetration indicated that a reaction-diffusion mechanism could explain the failure of these antibiotics to control biofilm infections (Stewart, 1996) and recent studies have shown that antimicrobial penetration times in biofilms differed over orders of magnitude due to reaction, absorption, and diffusion mechanisms, with reactive oxidants and cationic molecules associated with slower penetration (Stewart, 2015). The charge of polymers (Walters et al., 2003) and antibiotic-degrading enzymes such as β-lactamases present in the EPS matrix (Bagge et al., 2004; Armbruster et al., 2010) can lead to binding and/or deactivation of antibiotics. Dead cells within the biofilm can also attenuate antibiotic efficacy by binding antibiotics and diluting the stoichiometry of antibiotic molecules per cell (Mai-Prochnow et al., 2008). In the case of *P. aeruginosa*, anti-Pel and anti-Psl antibodies present in the airway bound to bacteria leading to biofilm aggregate formation accompanied by tobramycin tolerance (Colvin et al., 2011; Jennings et al., 2021; Morris et al., 2021). Aggregates also can harbor tolerant and “persister” bacterial sub-populations that can survive transient antibiotic therapy and subsequently regrow when treatment ends (Post, 2004; Conlon, 2013). Finally, aggregates can facilitate gene transfer of antimicrobial resistance genes. Bacteria in *P. aeruginosa* biofilms are competent for natural transformation of both genomic and plasmid DNA (Nolan et al., 2020) and biofilms can act as plasmid sinks, which can persist under non-selective conditions (Røder et al., 2021).

Notably, the physiological state of bacteria in biofilms crucially contributes to antimicrobial tolerance. The physiological heterogeneity of biofilm aggregates indicate that bacteria can be growing, stress-adapted, dormant, and metabolically active or inactive, all of which can impact antibiotic tolerance (Stewart, 2015; Crabbé et al., 2019). Interestingly, recent evidence suggests that while aggregated bacteria were present in acute as well as chronic infections in the lower respiratory tract, metabolic activity was more indicative of whether an infection was acute and presumably more treatable with antibiotics (Kolpen et al., 2022).

Common bacteria that infect the human airway and form biofilms include *Streptococcus pneumoniae*, Nontypeable *Haemophilus influenzae* (NTHi)*, Moraxella catarrhalis, Staphylococcus aureus*, and *Pseudomonas aeruginosa.* These have been defined largely by traditional culture-based microbiological diagnosis. However, culture-independent methods proved important in showing that culture missed a majority of infections in chronic otitis media, which could be identified by PCR methods (Post et al., 2004; Hall-Stoodley et al., 2006a). Culture-independent methods have since revealed that the airway microbiota are highly complex showing both aerobic and non-aerobic bacteria and fungi by genomic analysis, and are contingent on the airway epithelial cell type, anatomical structure, age, and disease status (Filkins and O'Toole, 2015; Man et al., 2017; Caverly and LiPuma, 2018; Welp and Bomberger, 2020). Genomic approaches however can be extremely sensitive detecting microbial DNA that may no longer be present at sampling. Other bacteria that are recognized as true pathogens, particularly in CF, include *Burkholderia cenocepacia* and nontuberculous mycobacteria (NTM) such as *Mycobacterium avium* and *Mycobacterium abscessus* (Jones, 2019). NTM have emerged as pathogens of concern due to the need for prolonged antibiotic therapy with *M. abscessus* particularly worrisome due to its antibiotic recalcitrance, along with antibiotic resistant *S. aureus* and *P. aeruginosa*. Preceding viral infection can impact airway bacterial infection (Armbruster et al., 2020) by impairing mucociliary clearance (Pittet, 2010) as well as impacting nutritional immunity by sequestering iron (Hendricks et al., 2016). Viral infection also can promote biofilm development by *P. aeruginosa* by the release of extracellular vesicles secreted from airway epithelial cells during respiratory viral infection (Hendricks et al., 2021).

## Upper respiratory tract infections involving biofilms - Chronic otitis media and chronic rhinosinusitis

It is unsurprising then that altered airway physiology and disrupted mucociliary clearance can predispose the host respiratory epithelial microenvironment to microbial aggregation. Otitis media, one of the most frequent illnesses in young children and the most common reason for antibiotic therapy or surgery in children in the US (Klein, 2000), is characterized by mucociliary dysfunction, blocked drainage of Eustachian tubes into the oropharynx, and defective clearance. While this occurs primarily in infants and young children due to immature facial-cranial anatomy, it is multifactorial (Bakaletz, 2010) and growing evidence suggests that preceding viral infection can play an important role (Brockson et al., 2012; Toone et al., 2020; Mokrzan et al., 2020; Kiedrowski and Bomberger, 2018; Hendricks et al., 2021). The middle ear epithelium consists of ciliated respiratory epithelial cells (**Figure 1**) and when mucociliary function is disrupted, bacteria in the normal oropharyngeal and nasopharyngeal microbiota can ascend the Eustachian tube to the middle ear mucosa (Bakaletz, 2010). The dominant bacterial opportunistic pathogens found in OM, *S. pneumoniae*, NTHi, and *M. catarrhalis*, typically fail to produce signs of infection or inflammation during colonization as commensal microbiota until compromising conditions result in bacterial access to the middle ear microenvironment. When middle ear mucosal biopsy samples recovered from children with either chronic or recurrent OM were evaluated using PCR of the effusion (if present), fluorescent *in situ* hybridization (FISH), and immunolabeling of bacteria together with confocal scanning laser microscopy (CSLM), S*. pneumoniae*, NTHi*, and M. catarrhalis* presented as biofilm aggregates on the middle ear mucosal epithelium of children with both OM with effusion and children with recurrent otitis media without effusion (Hall-Stoodley et al., 2006a). There was no evidence of adherent bacteria by 16S rRNA probes in non-OM biopsies. In contrast, further studies using both untargeted probes and pathogen-specific FISH probes demonstrated that OM-specific pathogens were found on the adenoid mucosal surface in both healthy and children with OM (Nistico et al., 2011).

In response to these bacterial otopathogens, middle ear epithelial cells produce predominantly gel-like mucins, MUC2, MUC5A, and MUC5B, which may be more effective in entrapping bacteria, but may also be more predisposed to blockage (Kershner et al., 2014). While MUC2 is a normal mucin of the gastrointestinal tract, it is associated with the airways in people with diseases such as CF and COPD. Infection leads to cytokine and mucin gene expression, inflammation, mucosal hyperplasia, effusion, and the infiltration of innate cells into the middle ear (Samuels et al., 2017). Mucin (MUC) gene polymorphism in children with chronic OM may also contribute to the inadequate host clearance of NTHi, *S. pneumoniae and M. catarrhalis* (Kerschner, 2007; Ubell et al., 2008).

*In vivo* studies using the chinchilla animal model of OM have demonstrated that NTHi infection can lead to large biofilm aggregates on the middle ear mucosa, mediated by type IV pili and extracellular DNA (Jurcisek and Bakaletz, 2007; Jurcisek et al., 2017; Novotny and Bakaletz, 2016). Importantly, this stagnant mucin-rich effusion can promote bacterial proliferation (Miyamoto and Bakaletz, 1996). Vaccination *via* a transcutaneous route in the chinchilla model increased NTHi-specific IgA in middle ear fluid and decreased NTHi biofilms (Novotny and Bakaletz, 2020). While *M. catarrhalis* also can be evaluated in chinchillas (Luke et al., 2007), *S. pneumoniae* is extremely virulent and biofilm infections are not easily interrogated in this animal model.

Chronic sinus infections similarly exhibit an abnormal accumulation of mucus associated with increased fluid pressure and dysfunctional drainage and clearance of mucus from the sinuses into the nasopharynx. Chronic rhinosinusitis (CRS) is a widespread infection of the upper airway affecting 11-13% of the general population in Europe and the US (Bose et al., 2016). CRS involves the mucosa lining the sinonasal cavity and is characterized by recurrent episodes of inflammation and chronic symptoms that include nasal obstruction, sinus pain, and rhinorrhea. The disease can manifest clinically as chronic rhinosinusitis without nasal polyps (CRSsNP) or with polyps (CRSwNP), which requires surgery. Several studies have evaluated CRS using endoscopic sinus surgery due to the persistent symptoms that can occur postoperatively (e.g., ongoing mucosal inflammation, and recurrent infections) and have provided evidence that bacterial biofilms play a role in the protracted mucosal inflammation in CRS (Sanderson et al., 2006; Psaltis et al., 2007; Sanclement et al., 2005; Prince et al., 2008; Foreman and Wormald, 2010; Singhal et al., 2010). Interestingly, different biofilm species are associated with different disease phenotypes. *H. influenzae* for example, is associated with patients with milder disease, while *S. aureus* was associated with more severe and recalcitrant disease (Prince et al., 2008; Foreman and Wormald, 2010; Singhal et al., 2011). *S. aureus* is an ubiquitous biofilm-forming pathogen that can asymptomatically colonize the upper respiratory tract and sinonasal cavities. In studies evaluating biopsy samples using FISH/CLSM and PCR from people with or without nasal polyps, surface-associated bacterial biofilm aggregates were present on 9/9 non-polypoidal sinonasal mucosa samples, including *S aureus* in 78%, *H. influenzae* in 33%, and *P. aeruginosa* in 33% of samples, but none of the non-CRS samples (Hayes et al., 2015). Interestingly In this study, *S. aureus* was not present on the epithelial surface of nasal polyps, but rather intracellularly in mast cells in the nasal epithelium. Follow-up studies with tissue explants indicated that *S. aureus* can be captured in extracellular traps and phagocytosed by mast cells and intracellular *S. aureus* promoted mast cell migration into the mucosal subepithelial layer, cell death, and the release of inflammatory mediators (Hayes et al., 2020). Notably, there is a high prevalence of CRS in individuals with CF and PCD, which may suggest a hereditary component together with multiple host genes that could further contribute to CRS (Bose et al., 2016).

## Lower respiratory tract infections

In lower airway biofilm infections such as in people with cystic fibrosis (CF), primary ciliary dyskinesia (PCD), or chronic obstructive pulmonary disease (COPD), mucociliary clearance is impeded by heavy sticky mucus and reduced airway surface liquid, by immotile or dysfunctional cilia, and/or by damage to the respiratory epithelium. Increased mucus production by goblet cells is a common feature of CF, PCD, and COPD (Davis and Wypych, 2021). These diseases were recently characterized as “muco-obstructive lung diseases” by Boucher to better describe their clinical presentation of diffuse mucus obstruction, dilation of airway walls, prolonged inflammation, and recurrent infection (Boucher, 2019). Muco-obstructive diseases in the lung therefore arise from different pathophysiological mechanisms that can involve defective epithelial cilia motility, ion transport and fluid homeostasis, or mucin secretion that results in the accumulation and stasis of mucus in airway compartments, which is not cleared and provides a microenvironment for persistent airflow obstruction, inflammation, and infection. In the examples discussed below, this can provide a microenvironment for the growth of bacteria and development of biofilm aggregates.

## Cystic fibrosis

CF is a life-threatening genetic disease more prevalent in people of European descent, although as improved molecular diagnostic methods identify CF, this disease is increasingly being found in non-European populations and in individuals with noncanonical presentations of CF and related disorders (Bell et al., 2020). Even with promising new therapeutic approaches to treat disease such as highly effective modulator therapy, a substantial population of people with CF (PwCF) continues to suffer considerable morbidity primarily due to lung infections with a select array of opportunistic pathogens (Cohen and Prince, 2012; Malhotra et al., 2019). CF is caused by mutations in the gene encoding the cystic fibrosis transmembrane conductance regulator (CFTR) ion channel, which transports chloride and bicarbonate to maintain osmotic balance across multiple epithelial surfaces. Mutations in CFTR can markedly disrupt the transport of chloride and bicarbonate into the airway epithelial lumen, which is normally counterbalanced by the epithelial sodium channel (ENac). Most mutations in CFTR result in increased water uptake by the epithelium together with dysregulated sodium reabsorption. In the lung this results in pathophysiology that leads to dehydration of the airway surface liquid (ASL), increased mucous viscosity, concomitant disruption of mucociliary clearance, mucus plugging or airway obstruction, inflammation, and infection that ultimately can decimate lung function (Matsui, 1998; Matsui et al., 2006; Cantin et al., 2015; Elborn, 2016; Malhotra et al., 2019). Studies also indicate that the ASL pH is lower in CF airways, which can lead to decreased host antimicrobial effectors in the airway (Pezzulo et al., 2012; Shah et al., 2016). Nonetheless, CF pathophysiology is multifactorial involving multiple deficits in host innate immunity and an airway microenvironment that is predisposed to hyperinflammation (for a comprehensive discussion of *P. aeruginosa* in CF see Malhotra et al., 2019). Histologically, large plugs of mucus, bacteria, and inflammatory cells can be present in CF airways along with hyperplasia of submucosal cells, which lead to chronic obstruction (Katkin, 2022). Thus, multiple deficits are present in the lungs of PwCF including decreased ciliary movement and mucus transport, decreased pH, abnormal sodium and chloride concentrations of the airway fluid, defective immune cell function by macrophages and neutrophils, and hyperinflammation (Cohen and Prince, 2012; Bruscia and Bonfield, 2016).

The secreted airway mucins (primarily MUC5AC, MUC5B) are extremely hydrophilic and create a reservoir whereby water is concentrated in the periciliary space to ensure that the ASL is sufficiently hydrated. CFTR plays a key role in providing the water necessary to balance the irrigation of the epithelial compartments by secreting chloride and directly or indirectly regulating sodium absorption. In CF changes to mucus viscoelasticity have been attributed to mucus hyper-secretion and changes to mucus composition, which impedes mucus clearance by both mucociliary clearance and cough clearance. Theoretical indices of the biophysical properties of mucus were developed that correlate viscoelastic properties of expectorated mucus with clearance to define predicted levels of clearance from the lung such as the mucociliary clearance index (MCI) and cough clearance index (CCI) (Lieleg et al., 2011; Gloag et al., 2018; Gloag et al., 2021). MCI predicts that elasticity of mucus correlated with improved mucociliary clearance by promoting efficient cilia beating (Lieleg et al., 2011; Zayas et al., 1990), while CCI predicts that mucus viscosity is correlated with improved clearance when the cohesive forces of the mucus gel are disrupted and expelled by cough (Zayas et al., 1990; Dasgupta and King, 1996).

Intriguingly, bacterial biofilms also display the biophysical characteristics of both viscoelastic solids and liquids (Peterson et al., 2015). Rheological analysis of biofilms indicated that mechanical stress can impact the microbial community structure and composition, which includes the EPS matrix and bacteria-bacteria interactions. Importantly, biofilm viscoelastic properties correlate with poor antimicrobial penetration into biofilms and play a role in protecting against mechanical and chemical challenges in numerous environments (Gloag et al., 2019). In CF the abnormal mucus accumulation and impaired clearance can form a niche that can be readily colonized by select bacteria that can form biofilm aggregates (Lam et al., 1980; Singh et al., 2000; Worlitzsch et al., 2002; Bjarnsholt et al., 2009; Cohen and Prince, 2012). Relevant to airway infections, the relationship between viscoelasticity and clearance has been studied with *P. aeruginosa* and with the NTM*, M. abscessus*, an emerging antibiotic-recalcitrant pathogen in individuals with CF, to examine if the viscoelastic properties of bacterial biofilms could impact the defined theoretically determined indices of mechanical clearance (Gloag et al., 2018; Gloag et al., 2021). Rheological measurements of biofilms of different variants of *P. aeruginosa* (mucoid and non-mucoid) and *M. abscessus* morphotypes (smooth or rough) were associated with significantly lower predicted clearance.

CF airway infections ensue from a circumscribed array of environmental pathogens as well as from opportunistic pathogens from the normal microbiota, which can grow in the airway mucus. Early in life, the airways of children with CF are colonized with *H. influenzae*, *S. pneumoniae*, and *M. catarrhalis* as well as *S. aureus* and *P. aeruginosa.* Pathogens can vary with age with *H. influenzae S. pneumoniae*, and *M. catarrhalis* decreasing past adolescence. Remarkably, infection with *S. aureus* and *P. aeruginosa* however was associated with neutrophil infiltration and airway deterioration even in infants that were deemed well, i.e., with no clinical signs and symptoms of exacerbation (Pillarisetti et al., 2011). Other organisms such as *B. cenocepacia*, *Stenotrophomonas maltophilia*, and *Achromobacter xylosoxidans* have remained important pathogens over the last 3 decades Cystic Fibrosis Foundation (2020). Notably, the prevalence of *S. aureus* decreases in older PwCF with a decreased prevalence of multidrug sensitive *S. aureus* (MSSR) and multidrug resistant *S. aureus* (MRSA), while *P. aeruginosa* increases together with an increased prevalence of multi-drug resistant strains. NTM, such as *M. avium* and *M. abscessus*, are increasing in prevalence in the general population and especially in PwCF, with the prevalence of NTM ranging between 10-14% (Floto et al., 2016; Matsuyama et al., 2018). Over the last ten years, *M. abscessus* has been increasing as a proportion of NTM and can be extremely challenging to treat due to its antibiotic resistance (CFF Patient Registry Annual Data Report, 2020). As with the respiratory tract in general, the advent of culture-independent microbiological evaluation of microbiota has been revealing. These studies have shown a wide diversity of microbiota in the CF lung comprised of multiple anaerobic bacteria, viruses and fungi that decrease with age and decreasing lung function, for review see (Filkins and O'Toole, 2015; Caverly and LiPuma, 2018).

Chronic cycles of pulmonary infection are a primary contributor to the morbidity and mortality associated with CF. CF was the first disease to be associated with *P. aeruginosa* mucoid strains and biofilm aggregates in the airway (Høiby, 1974: Lam et al., 1980). Decreased ciliary function and mucus transport leading to mucus stasis, along with higher acidity and abnormal sodium and chloride concentrations in the airway secretions provides a fertile microenvironmental niche for bacterial aggregation and growth. The CF airway epithelium consumes oxygen that can establish zones of low oxygen, which can be readily colonized by *P. aeruginosa*, where it can form biofilm aggregates (Worlitzsch et al., 2002). Multiple enzyme systems also allow *P. aeruginosa* to undergo remarkable physiological changes and adaptations to these microaerobic and anaerobic microenvironments present in the CF lung by using denitrification and fermentation (Alvarez-Ortega and Harwood, 2007; Schobert and Jahn, 2010). Nutritional immunity has also been proposed to explain why nutrients such as iron, which is needed for bacterial growth and metabolism, is regulated by the host to sequester free iron. Nutrient availability was examined during viral infection of airway epithelial cells (AEC) (Hendricks et al., 2016). Transferrin, a principal host iron-binding protein, was significantly increased on the apical surface of polarized AECs during virus infection and promoted *P. aeruginosa* biofilm development both *in vitro* and *in vivo*, suggesting that disruption of nutritional immunity during respiratory viral infection could lead to a favorable microenvironment for secondary bacterial infection.

Over time *P. aeruginosa* can undergo clonal selection and diversification in the lungs of PwCF. The most well-known example of diversification is the development of mucoidy in chronically infected CF lungs (Malhotra et al., 2018). Mucoid *P. aeruginosa* variants overproduce the exopolysaccharide alginate, a polymer of D-mannuronic and L-guluronic acid (Hogardt and Heesemann, 2010). Infection with mucoid *P. aeruginosa* is associated with antimicrobial recalcitrance, and in the host, increasing bronchiectasis, rapid decline in lung function, and increased mortality that likely reflects the complex pathoadaptation to the CF lung microenvironment (Smith et al., 2006; Malhotra et al., 2019). However, non-mucoid *P. aeruginosa* can also form aggregates, as in the hyper-biofilm forming *P. aeruginosa* rugose small-colony clinical variant (RSCV) (Pestrak et al., 2018). This aggregative phenotype promoted phagocyte evasion, stimulated neutrophil reactive oxygen species (ROS) production, and inflammatory cytokine production as well as enhanced tolerance to neutrophil-produced antimicrobials including H_2_O_2_ and the antimicrobial peptide LL-37. Interestingly LL-37 can contribute to *P. aeruginosa* mutagenesis and pathoadaptation (Limoli et al., 2014; Malhotra et al., 2018).

## Primary ciliary dyskinesia

Primary ciliary dyskinesia (PCD), called Kartagener’s syndrome in subset of PCD who also have situs inversus, is an autosomal recessive disorder that affects approximately 1 in 15,000 births. Unlike CF, the genetic mutations in PCD are polygenic and this genetic heterogenicity makes estimation of prevalence challenging (Knowles et al., 2013; Lucas and Leigh, 2014; Shapiro, 2016). PCD causes a spectrum of cilial ultrastructural and functional defects, including dyskinetic movement of respiratory cilia (Walker et al., 2012; Goutaki et al., 2016). At the molecular level the motile ciliary axoneme is arranged in a “9+2” configuration with nine peripheral microtubule doublets surrounding a central pair of single microtubules that together with ~250 other proteins including radial spokes and nexin links, maintain the axoneme structure. The inner and outer arms of the axoneme are formed by dynein complexes, which act as mechanochemical ATPases that generate the force required for ciliary beating (Leigh et al., 2009; Shapiro et al., 2016; Lucas et al., 2017). The radial spokes coordinate the synchronous pattern of ciliary beating and therefore mutations that affect the dynein arms or radial spokes can cause the ciliary movement to be dyskinetic or absent altogether, resulting in abnormal ciliary function and compromised mucociliary clearance. Consequently, people with PCD have recurrent and chronic sinopulmonary infection, chronic otitis media (OM), and progressive suppurative lung disease from a young age (Walker et al., 2017). Interestingly, while individuals with CF or individuals with PCD are both prone to upper respiratory tract infections, PwCF are predisposed to CRS, while people with PCD are susceptible to both chronic OM and CRS. Whether this is due to mucins or to the cilia in each compartment in each disease is unknown, however a study of the sinuses in people with PCD or with CF indicated considerable overlap regarding the microbiology (Møller et al., 2017).

To study the role of the surface epithelium in PCD, primary airway epithelial cells (AEC) can be grown at air liquid interface (ALI) to develop polarized well-differentiated ciliated epithelial mucosa. This model was used to compare the airway microenvironment from people with PCD and age-matched individuals without PCD to investigate the hypothesis that abnormal ciliary motility and low nitric oxide (NO) levels were permissive for biofilm aggregate formation with the most common PCD-associated pathogen, NTHi (Walker et al., 2017). Using image analysis and confocal microscopy the volume of bacterial aggregates was quantified and demonstrated that PCD AEC cultures with dysfunctional cilia developed significantly larger biofilm aggregates with significantly higher numbers of NTHi adherent to PCD epithelium. In contrast, non-PCD AEC cultures had significantly fewer adherent NTHi. Cytokine and LL37 production were comparable in the non-PCD and PCD cohorts. Surprisingly, however NO levels and expression of eNOS, iNOS, and nNOS were also comparable in both cohorts, suggesting abnormal ciliary motility was the principal defect associated with aggregate development. Nonetheless, an antibiotic combined with a NO-donor significantly increased killing of NTHi and reduced NTHi aggregates on PCD epithelium. In a separate study, NO-donor treatment significantly increased the susceptibility of *in vitro* NTHi biofilm to azithromycin, leading to a 10-fold reduction in viable bacteria compared to antibiotic alone (Collins, 2017). However, when NTHi biofilms were evaluated on primary respiratory epithelial cell cultures, treatment with both a NO-donor and azithromycin led to a 100-fold reduction in viable bacteria. Proteomic analyses suggested that exogenous NO increased expression of bacterial proteins involved in metabolic and transcriptional/translational functions and boosted azithromycin efficacy by modulating NTHi metabolic activity.

Infections with *P. aeruginosa* also increase morbidity in PCD (Alanin, 2015) and a recent study provided evidence of phenotypic and genotypic parallelism in *P. aeruginosa* evolution in infections in people with PCD or CF (Sommer et al., 2016). In this study over 80% of people with PCD had persistent clones of *P. aeruginosa* and genes from these clones were also found in persistent infections in CF airways, including genes conferring antibiotic resistance and associated with quorum sensing, motility, type III secretion, and mucoidy. These data suggest that pathoadaptive changes may be governed by similar selective forces such as the intensive antibiotic treatment and inflammatory responses to drive microbial diversification in airway infections.

## Chronic obstructive pulmonary disease

The progression of disease in chronic obstructive pulmonary disease (COPD) is similar to that of the other two muco-obstructive lung diseases discussed above. COPD is associated with mucus accumulation in the airway lumen and the infiltration of innate and adaptive inflammatory immune cells, which facilitate repair or remodeling processes that can result in thickened airway epithelial walls (Boucher, 2019). The persistent respiratory symptoms together with limited airflow in COPD includes the distinct clinical entities, emphysema, bronchiolitis, and chronic bronchitis (Ahearn et al., 2017). Emphysema and chronic bronchitis are the two most common conditions that contribute to COPD. These conditions often occur together and can vary in severity among individuals with COPD. Emphysema is characterized by damage to lung parenchyma and the collapse of alveoli resulting in substantially decreased surface area for the exchange of oxygen and CO_2_. Alpha-1 antitrypsin (AAT) deficiency is an established genetic cause of COPD, with those homozygous for the Z allele of the SERPINA1 gene, inheriting two copies of the protease inhibitor (Foreman et al., 2017). While people heterozygous for the gene encoding alpha-1 antitrypsin (AAT) deficiency who are cigarette smokers show a higher risk for COPD, the mechanism of the increased risk is unknown, and a recent study has suggested that people heterozygous for AAT may be a subset of COPD disease (Ghosh et al., 2022). Bronchiolitis and chronic bronchitis, on the other hand, are characterized by inflammation and fibrosis of the small airways or the accumulation of mucus due to goblet cell metaplasia and the increased secretion of mucus, respectively.

Although genetics likely play a role in a certain percentage of cases, COPD is linked principally to long-term exposure to toxins and irritants, most frequently in cigarette smoke (including secondary and tertiary smoke) (Sethi and Murphy, 2008; Ahearn et al., 2017). Up to a third of people with COPD however have never smoked and ~30-40% of cases are not associated with smoking, but rather are due to other causes such as air pollutants. While the literature indicates that outdoor air pollution is linked with increased COPD exacerbations and mortality, excessive morbidity and mortality among individuals with COPD associated with temperature extremes, suggest that climate change may have a deleterious impact on managing this disease (Hansel et al., 2016). Generally, mucociliary clearance and the epithelial barrier are both disrupted by persistent acute inflammatory responses, which exacerbate symptoms, contribute to airway inflammation, and hasten the advancement of diminished pulmonary function (Ahearn et al., 2017; Hogg et al., 2004; Agustí and Hogg, 2019). Notably, the thickened airway lumen can become obstructed with mucus and an influx of neutrophils and macrophages as well as adaptive immune cells, which also correlates with impaired IgA (Gohy et al., 2014). Pathogens involved in COPD exacerbations are similar to those in chronic OM and in PCD, with NTHi the most common bacterial cause of infection, accounting for nearly half of exacerbations in people with COPD (Ahearn et al., 2017). Interestingly, the acquisition of new NTHi strains may play a role in pathogenesis (Sethi and Murphy, 2008). As in chronic OM, NTHi exhibits pathoadaptation, including expressing adhesins that mediate adherence to host cells, invasion of epithelial cells, evasion of host immune cells and clearance, biofilm formation, and surface antigenic variation (Ahearn et al., 2017; Fernández-Calvet et al., 2021).

In a recent study comparing sputum samples from people with COPD, community acquired pneumonia, and CF sputum as a positive sample cohort exhibiting biofilm aggregates, bacterial aggregates were found to dominate over planktonic bacteria, regardless of whether the infection was defined as acute or chronic (Kolpen et al., 2022) . While this study suggests that aggregates may be more prevalent in lower airway infections than previously thought, only sputum samples were evaluated and since people with COPD are at higher risk of CAP, it is not clear if bronchiectasis plays a role in bacterial aggregation. Since the role of mucus in health appears to minimize bacterial aggregation, it would be interesting to compare mucin samples from these patient groups.

## Microenvironmental conditions that promote aggregation

The CF lung microenvironment clearly influences bacterial aggregate formation. As previously discussed, *P. aeruginosa* was found in bacterial aggregates enclosed in a matrix within O_2_-depleted mucus from PwCF, which were not adherent to the airway epithelium (Worlitzsch et al., 2002). The stagnant mucin-rich effusion that appears to promote bacterial proliferation has also been demonstrated in the chinchilla model of OM (Miyamoto and Bakaletz, 1997; Jurcisek and Bakaletz, 2007; Novotny and Bakaletz, 2016), and mucin (MUC) gene polymorphism in patients with chronic OM may contribute to the inadequate host clearance of bacteria (Kerschner, 2007; Ubell et al., 2008). Such microbial aggregates are better protected from host cells and immune effectors, such as phagocytosis, and killing by reactive oxygen species (ROS), neutrophil extracellular traps (NETs), and antimicrobial peptides (Mishra et al., 2012; Alhede, 2020b). Aggregated bacteria can also provoke resident macrophages to produce pro-inflammatory cytokines such as IL-6, IL-8, and tumor necrosis factor alpha (TNF-α), which results in the signaling and recruitment of innate immune cells such as circulating monocytes and neutrophils into the airway (Bhattacharya et al., 2018; Bhattacharya et al., 2020). Sustained infection with *P. aeruginosa* for example results in a hyperinflammatory microenvironment that can be accompanied by tissue destruction leading to cycles of more inflammatory cells (Bruscia and Bonfield, 2016; Moser et al., 2021). Better understanding the infectious microenvironment will provide important insights into the diverse ways that *P. aeruginosa* structure/function adapts to wounds, device-related infections, and innate immune cells (Bjarnsholt et al., 2022).

Despite longstanding evidence of biofilms in the context of the CF airway, how aggregates form *in vivo* continues to remain open to debate. Recently, the idea that bacterial aggregates may be simply agglutinated by host polymers has been explored. Conditions that promoted *P. aeruginosa* aggregation such as increased mucus density, also increased resistance to antibiotics, H_2_O_2_, and killing by neutrophils, but also reduced invasiveness (Staudinger et al., 2014). Interestingly, mutations that typically inhibit aggregation did not abrogate the ability of bacteria to aggregate in a gel-like microenvironment. Using mucin and eDNA to model the dominant polymers found in the CF airway, two biophysical mechanisms of aggregation, bridging and depletion aggregation, were explored (Secor et al., 2018). Both mechanisms resulted in passive agglutination or aggregation of bacteria and a well-characterized biofilm-forming activity by bacteria *per se* was not required. This study suggests that bacterial aggregates may be a default bacterial mode of growth in some infection sites. S-IgA may also contribute to passive agglutination of airway bacteria, although its role remains largely unexplored (Sánchez Montalvo et al., 2022). Importantly, *aggregated bacteria were more tolerant to killing by antibiotics*.

Another study indicates that *P. aeruginosa* aggregate formation in sputum was due to the mechanism of polymer bridging (Jennings et al., 2021). Extracellular matrix polysaccharides Pel and Psl were evaluated in CF sputum with specific antibodies using immunohistochemistry. Both Pel and Psl exopolysaccharides were present in sputum and aggregation was exopolysaccharide dependent. Specifically, Pel was positively charged at the lower pH typically present in the CF airway, and bound to eDNA, which both increased the tolerance of *P. aeruguinosa* aggregates to tobramycin and protected biofilm aggregates from DNase, a nuclease used to break down extracellular DNA present in the CF airway (Jennings et al., 2021). *P. aeruginosa* Psl was also evaluated using CF-specific isolates and Psl-specific antibody was elevated in PwCF who failed antibiotic eradication treatment compared to those who were successfully treated (Morris et al., 2021). Persistent CF *P. aeruginosa* isolates exhibited increased aggregation mediated by bridging aggregation by antibody binding to Psl. Aggregates also displayed increased tolerance to tobramycin. These reports indicate that the development of *P. aeruginosa* aggregates can occur in a microenvironment that reflects the expectorated mucus found in the CF airway microenvironment (sputum) and that aggregation confers a heightened protection of bacteria that is observed in biofilms. While other studies provide ample evidence of the interplay of pathophysiological effects of airway diseases and infectious stimuli, including biofilm formation, these studies underscore the importance of using the human microenvironment representative of specific infection. Further studies are needed to address novel therapeutics that target airway-associated biofilm infections.

## Modeling the human airway microenvironment

Few *in vivo* models for CF airway infections fully recapitulate the pathology of the human airway diseases. Even in the examples of *P. aeruginosa*, the most common pathogen that infects the lungs of PwCF, *in vivo* animal models fail to allow the development of chronic infections in the airway without inducing aggregation in bacteria using agarose before infection (Thomsen et al., 2021). In other animal models such as pigs or ferrets, which each show >90% amino acid identity with human CFTR, mutations have been developed, however these models are limited by cost and breeding limited to a few labs (Welsh et al., 2009; Keiser et al., 2015). Why mice with a disrupted CFTR gene exhibit vastly different airway host defenses compared with the human airway may be due to the pH of airway surface liquid compared with human airway models (Shah et al, 2016). Similar difficulties exist for other genetic diseases that impact pulmonary disease such as PCD. For upper respiratory infections such as otitis media and rhinosinusitis, animal models have also been developed, however they take considerable optimization and may be limited to certain pathogens. Even for NTHi, the most common pathogen in OM (and COPD), animal models are unable to evaluate long-term infection (Ahearn et al., 2017).

To address this general insufficiency of animal models to mimic human infection, *ex vivo* models with human primary cells or cell lines have been developed. One challenge in evaluating clinical specimens for biofilms involves defining an appropriate control group or cohort, since obtaining samples from people without disease may not be feasible. Another challenge is the difficulty of directly obtaining and demonstrating aggregated cells in matrix enclosed cell clusters on tissue samples from the infected cohort, even though this is a basic diagnostic criterion for biofilm infections (Parsek and Singh, 2003; Hall-Stoodley and Stoodley, 2009). New *ex vivo* and *in vivo* models of infection with biofilm pathogens that better recapitulate the human airway are needed to improve *in vivo* evaluation and provide important data to inform the development of better preclinical modeling for developing therapies that target the airways.

While a few models in animals have been developed that can study biofilm infections *in vivo* (Novotny et al., 2005; Bakaletz, 2012), biofilm infections often fail to be recapitulated in KO mouse models, particularly in airway infections. Other animal models, such as the pig and ferret, must also be gauged by how accurately they recapitulate human diseases. The biofilm research community needs to address that despite animal models being the preferred models to mechanistically study the *in vivo* host response to pathogens, further progress requires the development of better chronic infection models (Nguyen and Singh, 2006; Coenye et al., 2019).

To model a microenvironment specific to biofilms associated with infection, understanding the anatomical localization and fluid/tissue is crucial. This may include pH, oxygen partial pressure, relevant host proteins, mucins, eDNA and mechanics. One example is *in vitro* synthetic CF medium (SCFM2) that has been developed to mimic CF sputum. SCFM2 includes mucins, eDNA, and representative nutrients and mimics the growth of *P. aeruginosa* and other pathogens found in sputum from individuals with CF. This medium was used to quantitatively evaluate the microbial phenotypes that develop in CF (Cornforth et al., 2020). A three-dimensional model of native mucus using isolated mucin glycans has also been used to evaluate the effects of these complex structures on *P. aeruginosa* (Wheeler et al., 2019). While reconstituted mucins can be purified according to specific conditions, such as pH, to eliminate much of the variability of native mucins further analysis is nevertheless necessary to determine if critical functions are similar in native mucus (Wagner et al., 2018).

Progress is also being made in modeling the tissue microenvironment *ex vivo*. Tissue biopsies can be used (lung, middle ear epithelium, adenoids, sinus samples) to evaluate respiratory tract infections using PCR and *in situ* CSLM (Hall-Stoodley et al., 2006a; Bjarnsholt et al., 2009; Nistico et al., 2011; Hayes et al., 2015; Allan et al., 2016). However, since a major drawback is the difficulty in obtaining tissue biopsies, particularly from cohorts without disease, researchers have developed human airway epithelial cell models to better understand the behavior of biofilm pathogens with human tissues to determine rudimentary host responses that lead to biofilm aggregate formation in the airway microenvironment. As discussed above, this model was used to compare the airway microenvironment from people with PCD and age-matched individuals without PCD to investigate the hypothesis that abnormal ciliary motility and low nitric oxide (NO) levels were permissive for biofilm formation with NTHi (Walker et al., 2017).

CF epithelial cell cultures using CFTR ΔF508 CFBE41o- mutant polarized epithelial cells have been used to investigate the human CF airway epithelial microenvironment with the most common CF pathogen, *P. aeruginosa*, to better understand the persistent colonization of the CF airway with this pathogen (Moreau-Marquis et al., 2008). Recently a novel cell line Calu-3 was developed to compare wild type and deleted CFTR (Morrison et al., 2022). These cells differentiated into polarized epithelium at ALI and were used in a primary HBE cell model to evaluate the effects of CFTR mutation on mucus production and its biochemical characteristics. Although infection was not studied, this model was able to evaluate the impacts of CFTR modulator therapies in *in vitro* airway cell cultures. A drawback of cell co-culture models with *P. aeruginosa* remains its profound cytotoxicity making many of these studies suited to evaluating early colonization. However, cytotoxicity is pathogen-dependent, since NTHi and NTM appear to be well tolerated over several days (Walker et al., 2017; Matsuyama et al., 2018).

Although *ex vivo* cells of primary human airway epithelial cells do not completely replicate all the abnormalities of deficient CFTR or PCD, they can provide important pre-clinical information about the impact of treatments on epithelial cell function in people with these conditions as well as other specific patient cohorts and to better understand the epithelial response to microbes (Auster et al., 2019; Elborn, 2016; Walker et al., 2017; Matsuyama et al., 2018; Rayner et al., 2019). Moreover, ALI differentiated airway epithelial cultures recapitulate key aspects of epithelial host defense including coordinated ciliary beating, regulation of airway surface fluid height, CFTR-dependent Cl- and HCO3- efflux, production of the gel-forming mucins MUC5B and MUC5AC, and IgA pIgR in the context of COPD, making this model a gold standard for assessing cellular and microbiological responses to infection with and without anti-infective treatments (Gohy et al., 2014; Walker et al., 2017; Rayner et al., 2019; Zemke et. al., 2020). While these models lack the contribution of a functioning immune response that animal models provide, the failure of animal models to recapitulate key aspects of human airway diseases must be considered with respect to the airway. Using innate immune cells from people with lung diseases such as PwCF may also provide insights specific to host-bacterial interactions that better reiterate human host responses (Bruscia and Bonfield, 2016).

Other approaches involve recapitulating the microenvironment of disease by using SCFM2 compared to sputum samples. Notably, when *P. aeruginosa* was studied in SCFM2 *together with* a CF epithelial cell model, these models exhibited the greatest genome-wide accuracy relative to CF sputum of the laboratory models studied (Cornforth et al., 2020). Although SCFM2 and the CF epithelial cell model did not recapitulate some specific functional categories, their accuracy outperformed the animal models comparators. Accuracy was further enhanced by studying CF *P. aeruginosa* clinical isolates. This key study demonstrates that combining multiple laboratory approaches can improve the models used to generate and investigate microenvironment-specific hypotheses relevant to microbial biofilms.

## Summary

This review has focused on biofilm aggregates in the human airway. The reader is directed to reviews that further discuss airway cellular composition, defenses, innate immune function, and microbiota to keep up with the ongoing and rapidly developing research in these areas. Understanding how the airway microenvironment impacts the structure/function of biofilm aggregates is still unfolding and further research is required to address the unmet need regarding how biofilms can be better managed in respiratory tract infections. Modeling the appropriate microenvironment by using relevant host cells, clinical isolates, and environmental conditions will help to better recapitulate key facets of biofilm aggregate development in specific anatomical sites in the airway. Importantly, microbial aggregates do not have to exhibit large surface-attached structures to be considered a biofilm. *Ex vivo* studies indicate that aggregates can be as small as 5-25μm and still display antibiotic-tolerant phenotypes. The high prevalence of CRS in individuals with CF and PCD, and OM in people with PCD suggests a hereditary component together with multiple host genes that contribute to these diseases and warrant further studies to evaluate common genetic pathways that might offer insights into the risks of developing biofilm infections. On the microbial side, evaluating the biogeography of infections can lead to important insights for host adaptation, mutation, and pathogen evolution. Finally, antibiotic tolerance can be reversed or abrogated by dispersing aggregated microbes and further studies with translationally appropriate models have the potential to provide new insights into the mechanisms that lead to biofilm formation as well as to advance the evaluation of novel therapeutic targets to augment antibiotic efficacy against biofilm aggregates. As we gain a more complete understanding of the modeling biofilm aggregates, so too will we better understand how to mitigate them.

## Author contributions

LH-S conceived and wrote the review. KM edited and reviewed for clinical relevance and accuracy. All authors contributed to the article and approved the submitted version.

## Funding

This work was supported by CFF Pilot and Feasibility Grant HALLST18I0, CFF Research Grant HALLST18G0, and MCCOY19R0. This work was supported in part by the Cure CF Columbus Translational Core (C3TC). C3TC is supported by the Division of Pediatric Pulmonary Medicine, the Biopathology Center Core, and the Data Collaboration Team at Nationwide Children’s Hospital. Grant support provided by The Ohio State University Center for Clinical and Translational Science (National Center for Advancing Translational Sciences, Grant UL1TR002733) and by the Cystic Fibrosis Foundation (Research Development Program, Grant MCCOY19R0).

## Conflict of interest

The authors declare that the research was conducted in the absence of any commercial or financial relationships that could be construed as a potential conflict of interest.

## Publisher’s note

All claims expressed in this article are solely those of the authors and do not necessarily represent those of their affiliated organizations, or those of the publisher, the editors and the reviewers. Any product that may be evaluated in this article, or claim that may be made by its manufacturer, is not guaranteed or endorsed by the publisher.

## References

[B1] Agustí A. Hogg J. C. (2019). Update on the pathogenesis of chronic obstructive pulmonary disease. N Engl. J. Med. 381 (13), 1248–1256. doi: 10.1056/NEJMra1900475 31553836

[B2] Ahearn C. P. Gallo M. C. Murphy T. F. (2017). Insights on persistent airway infection by non-typeable haemophilus influenzae in chronic obstructive pulmonary disease. Pathog. Dis. 75 (4), ftx042. doi: 10.1093/femspd/ftx042 PMC5437125 28449098

[B3] Alanin M. C. Nielsen K. G. von Buchwald C. Skov M. Aanaes K. Høiby N.. (2015). A longitudinal study of lung bacterial pathogens in patients with primary ciliary dyskinesia. Clin. Microbiol. Infect. 21, 1093 e1091–1097. doi: 10.1016/j.cmi.2015.08.020 26341913

[B4] Alhede M. Alhede M. Qvortrup K. Kragh K. N. Jensen PØ Stewart P. S.. (2020a). The origin of extracellular DNA in bacterial biofilm infections *in vivo* . Pathog. Dis. 78 (2), ftaa018. doi: 10.1093/femspd/ftaa018 32196074 PMC7150582

[B5] Alhede M. Kragh K. N. Qvortrup K. Allesen-Holm M. van Gennip M. Christensen L. D.. (2011). Phenotypes of non-attached pseudomonas aeruginosa aggregates resemble surface attached biofilm. PloS One 6 (11), e27943. doi: 10.1371/journal.pone.0027943 22132176 PMC3221681

[B6] Alhede M. Lorenz M. Fritz B. G. Jensen PØ Ring H. C. Bay L.. (2020b). Bacterial aggregate size determines phagocytosis efficiency of polymorphonuclear leukocytes. Med. Microbiol. Immunol. 209 (6), 669–680. doi: 10.1007/s00430-020-00691-1 32880037 PMC7568703

[B7] Allan R. N. Kelso M. J. Rineh A. Yepuri N. R. Feelisch M. Soren O.. (2017). Cephalosporin-NO-donor prodrug PYRRO-C3D shows β-lactam-mediated activity against streptococcus pneumoniae biofilms. Nitric. Oxide 65, 43–49. doi: 10.1016/j.niox.2017.02.006 28235635

[B8] Allan R. N. Morgan S. Brito-Mutunayagam S. Skipp P. Feelisch M. Hayes S. M.. (2016). Low concentrations of nitric oxide modulate streptococcus pneumoniae biofilm metabolism and antibiotic tolerance. Antimicrob. Agents Chemother. 60 (4), 2456–2466. doi: 10.1128/AAC.02432-15 26856845 PMC4808185

[B9] Allesen-Holm M. Barken K. B. Yang L. Klausen M. Webb J. S. Kjelleberg S.. (2006). A characterization of DNA release in pseudomonas aeruginosa cultures and biofilms. Mol. Microbiol. 59, 1114–1128. doi: 10.1111/j.1365-2958.2005.05008.x 16430688

[B10] Alvarez-Ortega C. Harwood C. S. (2007). Responses of pseudomonas aeruginosa to low oxygen indicate that growth in the cystic fibrosis lung is by aerobic respiration. Mol. Microbiol. 65 (1), 153–165. doi: 10.1111/j.1365-2958.2007.05772.x 17581126 PMC4157922

[B11] Armbruster C. R. Coenye T. Touqui L. Bomberger J. M. (2020). Interplay between host-microbe and microbe-microbe interactions in cystic fibrosis. J. Cyst Fibros. 19 Suppl 1 (Suppl 1), S47–S53. doi: 10.1016/j.jcf.2019.10.015 31685398 PMC7035965

[B12] Armbruster C. E. Hong W. Pang B. Weimer K. E. Juneau R. A. Turner J.. (2010). Indirect pathogenicity of haemophilus influenzae and moraxella catarrhalis in polymicrobial otitis media occurs *via* interspecies quorum signaling. mBio. 1 (3), e00102–e00110. doi: 10.1128/mBio.00102-10 20802829 PMC2925075

[B13] Auster L. Sutton M. Gwin M. C. Nitkin C. Bonfield T. L. (2019). Optimization of *In vitro* mycobacterium avium and mycobacterium intracellulare growth assays for therapeutic development. Microorganisms 7 (2), 42. doi: 10.3390/microorganisms7020042 PMC6406338 30717247

[B14] Bachofen H. Wilson T. A. (1997). “Micromechanics of the acinus and alveolar walls,” in The lung: scientific foundations, 2nd Ed. Eds. Crystal R. G. West J. B. (Philadelphia, Pa: Lippincott-Raven Publishers), 1159–1167.

[B15] Bagge N. Hentzer M. Andersen J. B. Ciofu O. Givskov M. Hoiby N. (2004). Dynamics and spatial distribution of β-lactamase expression in pseudomonas aeruginosa biofilms. Antimicrob. Agents Chemother. 48 (4), 1168–1174. doi: 10.1128/AAC.48.4.1168-1174.2004 15047517 PMC375278

[B16] Bakaletz L. O. (2010). Immunopathogenesis of polymicrobial otitis media. J. Leukoc. Biol. 87 (2), 213–222. doi: 10.1189/jlb.0709518 19843575 PMC2812561

[B17] Bakaletz L. O. (2012). Bacterial biofilms in the upper airway - evidence for role in pathology and implications for treatment of otitis media. Paediatr. Respir. Rev. 13 (3), 154–159. doi: 10.1016/j.prrv.2012.03.001 22726871 PMC3509202

[B18] Bell S. C. Mall M. A. Gutierrez H. Macek M. Madge S. Davies J. C.. (2020). The future of cystic fibrosis care: a global perspective. Lancet Respir. Med. 8 (1), 65–124. doi: 10.1016/S2213-2600(19)30337-6 31570318 PMC8862661

[B19] Bhattacharya M. Berends E. T. M. Chan R. Schwab E. Roy S. Sen C. K.. (2018). Staphylococcus aureus biofilms release leukocidins to elicit extracellular trap formation and evade neutrophil-mediated killing. Proc. Natl. Acad. Sci. U.S.A. 115 (28), 7416–7421. doi: 10.1073/pnas.1721949115 29941565 PMC6048508

[B20] Bhattacharya M. Berends E. T. M. Zheng X. Hill P. J. Chan R. Torres V. J.. (2020). Leukocidins and the nuclease nuc prevent neutrophil-mediated killing of staphylococcus aureus biofilms. Infect. Immun. 88 (10), e00372–e00320. doi: 10.1128/IAI.00372-20 32719153 PMC7504955

[B21] Bjarnsholt T. Jensen P.Ø. Fiandaca M. J. Pedersen J. Hansen C. R. Andersen C. B.. (2009). Pseudomonas aeruginosa biofilms in the respiratory tract of cystic fibrosis patients. Pediatr. Pulmonol. 44, 547–558. doi: 10.1002/ppul.21011 19418571

[B22] Bjarnsholt T. Whiteley M. Rumbaugh K. P. Stewart P. S. Jensen PØ Frimodt-Møller N. (2022). The importance of understanding the infectious microenvironment. Lancet Infect. Dis. 22 (3), e88–e92. doi: 10.1016/S1473-3099(21)00122-5 34506737 PMC9190128

[B23] Boles B. R. Singh P. K. (2008). Endogenous oxidative stress produces diversity and adaptability in biofilm communities. Proc. Natl. Acad. Sci. U.S.A. 105 (34), 12503–12508. doi: 10.1073/pnas.0801499105 18719125 PMC2527941

[B24] Boles B. R. Thoendel M. Singh P. K. (2004). Self-generated diversity produces "insurance effects" in biofilm communities. Proc. Natl. Acad. Sci. U.S.A. 101 (47), 16630–16635. doi: 10.1073/pnas.0407460101 15546998 PMC528905

[B25] Bose S. Grammer L. C. Peters A. T. (2016). Infectious chronic rhinosinusitis. J. Allergy Clin. Immunol. Pract. 4 (4), 584–589. doi: 10.1016/j.jaip.2016.04.008 27393772 PMC4939240

[B26] Boucher R. C. (2019). Muco-obstructive lung diseases. N Engl. J. Med. 380 (20), 1941–1953. doi: 10.1056/NEJMra1813799 31091375

[B27] Brockson M. E. Novotny L. A. Jurcisek J. A. McGillivary G. Bowers M. R. Bakaletz L. O. (2012). Respiratory syncytial virus promotes moraxella catarrhalis-induced ascending experimental otitis media. PloS One 7 (6), e40088. doi: 10.1371/journal.pone.0040088 22768228 PMC3387005

[B28] Bruscia E. M. Bonfield T. L. (2016). Cystic fibrosis lung immunity: The role of the macrophage. J. Innate Immun. 8 (6), 550–563. doi: 10.1159/000446825 27336915 PMC5089923

[B29] Button B. Cai L.-H. Ehre C. Kesimer M. Hill D. B. Sheehan J. K.. (2012). A periciliary brush promotes the lung health by separating the mucus layer from airway epithelia. Science 337, 937–941. doi: 10.1126/science.1223012 22923574 PMC3633213

[B30] Byrd M. S. Pang B. Mishra M. Swords W. E. Wozniak D. J. (2010). The pseudomonas aeruginosa exopolysaccharide psl facilitates surface adherence and NF-kappaB activation in A549 cells. MBio 1, e00140–e00110. doi: 10.1128/mBio.00140-10 20802825 PMC2925078

[B31] Cantin A. M. Hartl D. Konstan M. W. Chmiel J. F. (2015). Inflammation in cystic fibrosis lung disease: Pathogenesis and therapy. J. Cyst. Fibros. 14, 419–430. doi: 10.1016/j.jcf.2015.03.003 25814049

[B32] Caverly L. J. LiPuma J. J. (2018). Cystic fibrosis respiratory microbiota: unraveling complexity to inform clinical practice. Expert Rev. Respir. Med. 12 (10), 857–865. doi: 10.1080/17476348.2018.1513331 30118374 PMC6287746

[B33] Chi E. Mehl T. Nunn D. Lory S. (1991). Interaction of pseudomonas aeruginosa with A549 pneumocyte cells. Infect. Immun. 59, 822–828. doi: 10.1128/iai.59.3.822-828.1991 1671777 PMC258333

[B34] Clary G. Sasindran S. J. Nesbitt N. Mason L. Cole S. Azad A. (2018). Mycobacterium abscessus smooth and rough morphotypes form antimicrobial-tolerant biofilm phenotypes but are killed by acetic acid. Antimicrob Agents Chemother 62 (3), e01782-17. doi: 10.1128/AAC.01782-17 29311080 PMC5826145

[B35] Coenye T. Kjellerup B. Stoodley P. Bjarnsholt T. 2019 Biofilm Bash Participants (2019). The future of biofilm research - report on the '2019 biofilm bash'. Biofilm 2, 100012. doi: 10.1016/j.bioflm.2019.100012 33447799 PMC7798458

[B36] Cohen T. Prince A. (2012). Cystic fibrosis: a mucosal immunodeficiency syndrome. Nat. Med. 18, 509–519. doi: 10.1038/nm.2715 22481418 PMC3577071

[B37] Collin A. M. Lecocq M. Detry B. Carlier F. M. Bouzin C. de Sany P.. (2021). Loss of ciliated cells and altered airway epithelial integrity in cystic fibrosis. J. Cyst Fibros. 20 (6), e129–e139. doi: 10.1016/j.jcf.2021.09.019 34657818

[B38] Collins S. A. Kelso M. J. Rineh A. Yepuri N. R. Coles J. Jackson C. L.. (2017). Cephalosporin-3'-Diazeniumdiolate NO donor prodrug PYRRO-C3D enhances azithromycin susceptibility of nontypeable haemophilus influenzae biofilms. Antimicrob. Agents Chemother. 61 (2), e02086–e02016. doi: 10.1128/AAC.02086-16 27919896 PMC5278716

[B39] Colvin K. M. Gordon V. D. Murakami K. Borlee B. R. Wozniak D. J. Wong G. C. L.. (2011). The pel polysaccharide can serve a structural and protective role in the biofilm matrix of pseudomonas aeruginosa. PloS Pathog. 7, e1001264. doi: 10.1371/journal.ppat.1001264 21298031 PMC3029257

[B40] Conlon B. P. Nakayasu E. S. Fleck L. E. LaFleur M. D. Isabella V. M. Coleman K.. (2013). Activated ClpP kills persisters and eradicates a chronic biofilm infection. Nature 503 (7476), 365–370. doi: 10.1038/nature12790 24226776 PMC4031760

[B41] Cornforth D. M. Diggle F. L. Melvin J. A. Bomberger J. M. Whiteley M. (2020). Quantitative framework for model evaluation in microbiology research using pseudomonas aeruginosa and cystic fibrosis infection as a test case. mBio. 11 (1), e03042-19. doi: 10.1128/mBio.03042-19 31937646 PMC6960289

[B42] Crabbé A. Jensen PØ Bjarnsholt T. Coenye T. (2019). Antimicrobial tolerance and metabolic adaptations in microbial biofilms. Trends Microbiol. 27 (10), 850–863. doi: 10.1016/j.tim.2019.05.003 31178124

[B43] Crowther J. E. Schlesinger L. S. (2006). Endocytic pathway for surfactant protein a in human macrophages: binding, clathrin-mediated uptake, and trafficking through the endolysosomal pathway. Am. J. Physiol. Lung Cell Mol. Physiol. 290 (2), L334–L342. doi: 10.1152/ajplung.00267.2005 16169899

[B44] Cystic Fibrosis Foundation (2020). Cystic fibrosis foundation patient registry 2020 annual data report. Bethesda, MD. Available at: https://www.cff.org/sites/default/files/2021-11/Patient-Registry-Annual-Data-Report.pdf.sites/default/files/2021-11/Patient-Registry-Annual-Data-Report.pdf.

[B45] Dasgupta B. King M. (1996). Reduction in viscoelasticity in cystic fibrosis sputum *in vitro* using combined treatment with nacystelyn and rhDNase. Pediatr. Pulmonol. 22, 161–166. doi: 10.1002/(SICI)1099-0496(199609)22:3<161::AID-PPUL4>3.0.CO;2-S 8893254

[B46] Davis J. D. Wypych T. P. (2021). Cellular and functional heterogeneity of the airway epithelium. Mucosal Immunol. 14 (5), 978–990. doi: 10.1038/s41385-020-00370-7 33608655 PMC7893625

[B47] Dean C. H. Snelgrove R. J. (2018). New rules for club development: New insights into human small airway epithelial club cell ontogeny and function. Am. J. Respir. Crit. Care Med. 198 (11), 1355–1356. doi: 10.1164/rccm.201805-0925ED 29877729 PMC6290947

[B48] de Fays C. Carlier F. M. Gohy S. Pilette C. (2022). Secretory immunoglobulin a immunity in chronic obstructive respiratory diseases. Cells 11 (8), 1324. doi: 10.3390/cells11081324. 35456002 PMC9027823

[B49] Elborn J. S. (2016). Cystic fibrosis. Lancet 388 (10059), 2519–2531. doi: 10.1016/S0140-6736(16)00576-6 27140670

[B50] Ferguson J. S. Schlesinger L. S. (2000). Pulmonary surfactant in innate immunity and the pathogenesis of tuberculosis. Tuber Lung Dis. 80 (4-5), 173–184. doi: 10.1054/tuld.2000.0242 11052906

[B51] Fernández-Calvet A. Euba B. Gil-Campillo C. Catalan-Moreno A. Moleres J. Martí S.. (2021). Phase variation in HMW1A controls a phenotypic switch in haemophilus influenzae associated with pathoadaptation during persistent infection. mBio 12, e00789–e00721. doi: 10.1128/mBio.00789-21 PMC8262952 34154422

[B52] Filkins L. M. O'Toole G. A. (2015). Cystic fibrosis lung infections: Polymicrobial, complex, and hard to treat. PloS Pathog. 11 (12), e1005258. doi: 10.1371/journal.ppat.1005258 26719892 PMC4700991

[B53] Floto R. A. Olivier K. N. Saiman L. Daley C. L. Herrmann J. L. Nick J. A.. (2016). US Cystic fibrosis foundation and European cystic fibrosis society. consensus recommendations for the management of non-tuberculous mycobacteria in individuals with cystic fibrosis: executive summary. Thorax 71 (1), 88–90. doi: 10.1136/thoraxjnl-2015-207983 26678435 PMC4717423

[B54] Foreman A. Psaltis A. J. Tan L. W. Wormald P. J. (2009). Characterization of bacterial and fungal biofilms in chronic rhinosinusitis. Am. J. Rhinol. Allergy 23 (6), 556–561. doi: 10.2500/ajra.2009.23.3413 19958600

[B55] Foreman M. G. Wilson C. DeMeo D. L. Hersh C. P. Beaty T. H. Cho M. H.. (2017). Genetic epidemiology of COPD (COPDGene) investigators *. alpha-1 antitrypsin PiMZ genotype is associated with chronic obstructive pulmonary disease in two racial groups. Ann. Am. Thorac. Soc 14 (8), 1280–1287. 28380308 10.1513/AnnalsATS.201611-838OC PMC5566271

[B56] Foreman A. Wormald P. J. (2010). Different biofilms, different disease? a clinical outcomes study. Laryngoscope 120 (8), 1701–1706. doi: 10.1002/lary.21024 20641074

[B57] Fux C. A. Wilson S. Stoodley P. (2004). Detachment characteristics and oxacillin resistance of staphyloccocus aureus biofilm emboli in an *in vitro* catheter infection model. J. Bacteriol. 186 (14), 4486–4491. doi: 10.1128/JB.186.14.4486-4491.2004 15231780 PMC438612

[B58] Gakhar L. Bartlett J. A. Penterman J. Mizrachi D. Singh P. K. Mallampalli R. K.. (2010). PLUNC is a novel airway surfactant protein with anti-biofilm activity. PloS One 5 (2), e9098. doi: 10.1371/journal.pone.0009098 20161732 PMC2817724

[B59] Ghosh M. Brechbuhl H. M. Smith R. W. Li B. Hicks D. A. Titchner T.. (2011). Context-dependent differentiation of multipotential keratin 14-expressing tracheal basal cells. Am. J. Respir. Cell Mol. Biol. 45 (2), 403–410. doi: 10.1165/rcmb.2010-0283OC 21131447 PMC3175566

[B60] Ghosh A. J. Hobbs B. D. Moll M. Saferali A. Boueiz A. Yun J. H.. (2022). Alpha-1 antitrypsin MZ heterozygosity is an endotype of chronic obstructive pulmonary disease. Am. J. Respir. Crit. Care Med. 205 (3), 313–323. doi: 10.1164/rccm.202106-1404OC 34762809 PMC8886988

[B61] Ghosh M. Smith R. W. Runkle C. M. Hicks D. A. Helm K. M. Reynolds S. D. (2013). Regulation of trachebronchial tissue-specific stem cell pool size. Stem Cells 31 (12), 2767–2778. doi: 10.1002/stem.1440 23712882 PMC3844014

[B62] Gloag E. S. Fabbri S. Wozniak D. J. Stoodley P. (2019). Biofilm mechanics: Implications in infection and survival. Biofilm 2, 100017. doi: 10.1016/j.bioflm.2019.100017 33447803 PMC7798440

[B63] Gloag E. S. German G. K. Stoodley P and Wozniak D. J. (2018). Viscoelastic properties of pseudomonas aeruginosa variant biofilms. Sci. Rep. 8, 9691. doi: 10.1038/s41598-018-28009-5 29946126 PMC6018706

[B64] Gloag E. S. Wozniak D. J. Stoodley P. Hall-Stoodley L. (2021). Mycobacterium abscessus biofilms have viscoelasticity properties which may contribute to their recalcitrance in chronic pulmonary infections. Sci. Rep. 11 (1), 1–8. doi: 10.1038/s41598-021-84525-x 33658597 PMC7930093

[B65] Gohy S. T. Detry B. R. Lecocq M. Bouzin C. Weynand B. A. Amatngalim G. D.. (2014). Polymeric immunoglobulin receptor down-regulation in chronic obstructive pulmonary disease. persistence in the cultured epithelium and role of transforming growth factor-β. Am. J. Respir. Crit. Care Med. 190 (5), 509–521. doi: 10.1164/rccm.201311-1971OC 25078120

[B66] Goutaki M. Meier A. B. Halbeisen F. S. Lucas J. S. Dell S. D. Maurer E.. (2016). Clinical manifestations in primary ciliary dyskinesia: systematic review and meta-analysis. Eur. Respir. J. 48 (4), 1081–1095. doi: 10.1183/13993003.00736-2016 27492829

[B67] Hall-Stoodley L. Costerton J. W. Stoodley P. (2004). Bacterial biofilms: from the natural environment to infectious diseases. Nat. Rev. Microbiol. 2 (2), 95–108. doi: 10.1038/nrmicro821 15040259

[B68] Hall-Stoodley L. Hu F. Z. Gieseke A. Nistico L. Nguyen D. Hayes J.. (2006a). Direct detection of bacterial biofilms on the middle-ear mucosa of children with chronic otitis media. JAMA 296 (2), 202–211. doi: 10.1001/jama.296.2.202 16835426 PMC1885379

[B69] Hall-Stoodley L. Nistico L. Sambanthamoorthy K. Dice B. Nguyen D. Mershon W. J.. (2008). Characterization of biofilm matrix, degradation by DNase treatment and evidence of capsule downregulation in streptococcus pneumoniae clinical isolates. BMC Microbiol. 8, 173. doi: 10.1186/1471-2180-8-173 18842140 PMC2600794

[B70] Hall-Stoodley L. Stoodley P. (2009). Evolving concepts in biofilm infections. Cell Microbiol. 11 (7), 1034–1043. doi: 10.1111/j.1462-5822.2009.01323.x 19374653

[B71] Hall-Stoodley L. Stoodley P. Kathju S. Høiby N. Moser C. Costerton J. W.. (2012). Towards diagnostic guidelines for biofilm-associated infections. FEMS Immunol. Med. Microbiol. 65 (2), 127–145. doi: 10.1111/j.1574-695X.2012.00968.x 22469292

[B72] Hall-Stoodley L. Watts G. Crowther J. E. Balagopal A. Torrelles J. B. Robison-Cox J.. (2006b). Mycobacterium tuberculosis binding to human surfactant proteins a and d, fibronectin, and small airway epithelial cells under shear conditions. Infect. Immun. 74 (6), 3587–3596. doi: 10.1128/IAI.01644-05 16714591 PMC1479241

[B73] Hansel N. N. McCormack M. C. Kim V. (2016). The effects of air pollution and temperature on COPD. COPD 13 (3), 372–379. doi: 10.3109/15412555.2015.1089846 26683097 PMC4878829

[B74] Harkema J. R. Nikula K. J. Haschek W. M. (2018). “Respiratory system,” in Fundamentals of toxicologic pathology (Third edition). Eds. Wallig M. A. Haschek W. M. Rousseaux C. G. Bolon B. (London, United Kingdom: Academic Press), 351–393, ISBN: ISBN 9780128098417.

[B75] Häußler S. Ziegler I. Löttel A. Götz F. V. Rohde M. Wehmhöhner D.. (2003). Highly adherent small-colony variants of pseudomonas aeruginosa in cystic fibrosis lung infection. J. Med. Microbiol. 52 (Pt 4), 295–301. doi: 10.1099/jmm.0.05069-0 12676867

[B76] Hayes S. M. Biggs T. C. Goldie S. P. Harries P. G. Walls A. F. Allan R. N.. (2020). Staphylococcus aureus internalization in mast cells in nasal polyps: Characterization of interactions and potential mechanisms. J. Allergy Clin. Immunol. 145 (1), 147–159. doi: 10.1016/j.jaci.2019.06.013 31254531

[B77] Hayes S. M. Howlin R. Johnston D. A. Webb J. S. Clarke S. C. Stoodley P.. (2015). Intracellular residency of staphylococcus aureus within mast cells in nasal polyps: A novel observation. J. Allergy Clin. Immunol. 135 (6), 1648–1651. doi: 10.1016/j.jaci.2014.12.1929 25680455

[B78] Hendricks M. R. Lane S. Melvin J. A. Ouyang Y. Stolz D. B. Williams J. V.. (2021). Extracellular vesicles promote trans-kingdom nutrient transfer during viral-bacterial co-infection. Cell Rep. 34 (4), 108672. doi: 10.1016/j.celrep.2020.108672 33503419 PMC7918795

[B79] Hendricks M. R. Lashua L. P. Fischer D. K. Flitter B. A. Eichinger K. M. Dur- bin J. E.. (2016). Respiratory syncytial virus infection enhances pseudomonas aeruginosa biofilm growth through dysregulation of nutritional immunity. Proc. Natl. Acad. Sci. U.S.A. 113, 1642–1647. doi: 10.1073/pnas.1516979113 26729873 PMC4760822

[B80] Hill D. B. Button B. Rubinstein M. Boucher R. C. (2022). Physiology and pathophysiology of human airway mucus. Physiol. Rev. doi: 10.1152/physrev.00004.2021 PMC9665957 35001665

[B81] Høiby N. (1974). Pseudomonas aeruginosa infection in cystic fibrosis. relationship between mucoid strains of pseudomonas aeruginosa and the humoral immune response. Acta Pathol. Microbiol. Scand. Sect B 82, 551–558. 4213330

[B82] Hogardt M. Heesemann J. (2010). Adaptation of pseudomonas aeruginosa during persistence in the cystic fibrosis lung. Int. J. Med. Microbiol. 300 (8), 557–562. doi: 10.1016/j.ijmm.2010.08.008 20943439

[B83] Hogg J. C. Chu F. Utokaparch S. Woods R. Elliott W. M. Buzatu L.. (2004). The nature of small-airway obstruction in chronic obstructive pulmonary disease. N Engl. J. Med. 350 (26), 2645–2653. doi: 10.1056/NEJMoa032158 15215480

[B84] Hong K. U. Reynolds S. D. Watkins S. Fuchs E. Stripp B. R.. (2004). The nature of small-airway obstruction in chronic obstructive pulmonary disease. N Engl. J. Med. 164 (2), 577–88. doi: 10.1016/S0002-9440(10)63147-1 15215480

[B85] Howlin R. P. Cathie K. Hall-Stoodley L. Corne- lius V. Duignan C. Allan R. N.. (2017). Low-dose nitric oxide as targeted anti-biofilm adjunctive therapy to treat chronic pseudomonas aeruginosa infection in cystic fibrosis. Mol. Ther. 25 (9), 2104–2116. doi: 10.1016/j.ymthe.2017.06.021 28750737 PMC5589160

[B86] Irie Y. Borlee B. R. O’Connor J. R. Hill P. J. Harwood C. S. Wozniak D. J.. (2012). Self-produced exopolysaccharide is a signal that stimulates biofilm formation in pseudomonas aeruginosa. Proc. Natl. Acad. Sci. U.S.A. 109, 20632–20636. doi: 10.1073/pnas.1217993109 23175784 PMC3528562

[B87] Jefferson K. K. Goldmann D. A. Pier G. B. (2005). Use of confocal microscopy to analyze the rate of vancomycin penetration through staphylococcus aureus biofilms. Antimicrob. Agents Chemother. 49 (6), 2467–2473. doi: 10.1128/AAC.49.6.2467-2473.2005 15917548 PMC1140491

[B88] Jennings L. K. Dreifus J. E. Reichhardt C. Storek K. M. Secor P. R. Wozniak D. J.. (2021). Pseudomonas aeruginosa aggregates in cystic fibrosis sputum produce exopolysaccharides that likely impede current therapies. Cell Rep. 34 (8), 108782. doi: 10.1016/j.celrep.2021.108782 33626358 PMC7958924

[B89] Jennings L. K. Storek K. M. Ledvina H. E. Coulon C. Marmont L. S. Sadovskaya I.. (2015). Pel is a cationic exopolysaccharide that cross-links extracellular DNA in the pseudomonas aeruginosa biofilm matrix. Proc. Natl. Acad. Sci. U.S.A. 112, 11353–11358. doi: 10.1073/pnas.1503058112 26311845 PMC4568648

[B90] Jones A. M.. (2019). Which pathogens should we worry about? Paediatr Respir Rev. 31 (15), 17. doi: 10.1016/j.prrv.2019.02.007 30967346

[B91] Jurcisek J. A. Bakaletz L. O. (2007). Biofilms formed by nontypeable haemophilus influenzae *in vivo* contain both double-stranded DNA and type IV pilin protein. J. Bacteriol. 189 (10), 3868–3875. doi: 10.1128/JB.01935-06 17322318 PMC1913342

[B92] Jurcisek J. A. Brockman K. L. Novotny L. A. Goodman S. D. Bakaletz L. O. (2017). Nontypeable haemophilus influenzae releases DNA and DNABII proteins *via* a T4SS-like complex and ComE of the type IV pilus machinery. Proc. Natl. Acad. Sci. U.S.A. 114 (32), E6632–E6641. doi: 10.1073/pnas.1705508114 28696280 PMC5559034

[B93] Katkin J. P. (2022) Cystic fibrosis: Clinical manifestations of pulmonary disease. Available at: https://www.uptodate.com/contents/cystic-fibrosis-clinical-manifestations-and-diagnosis (Accessed 1.28.22).

[B94] Keiser N. W. Birket S. E. Evans I. A. Tyler S. R. Crooke A. K. Sun X.. (2015). Defective innate immunity and hyperinflammation in newborn cystic fibrosis transmembrane conductance regulator-knockout ferret lungs. Am. J. Respir. Cell Mol. Biol. 52 (6), 683–694. doi: 10.1165/rcmb.2014-0250OC 25317669 PMC4491130

[B95] Kerschner J. E. (2007). Mucin gene expression in human middle ear epithelium. Laryngoscope 117 (9), 1666–1676. doi: 10.1097/MLG.0b013e31806db531 17667140

[B96] Kerschner J. E. Hong W. Khampang P. Johnston N. (2014). Differential response of gel-forming mucins to pathogenic middle ear bacteria. Int J Pediatr Otorhinolaryngol 78 (8), 1668–73. doi: 10.1016/j.ijporl.2014.05.037 PMC4104932 24958163

[B97] Kiedrowski M. R. Bomberger J. M. (2018). Viral-bacterial Co-infections in the cystic fibrosis respiratory tract. Front. Immunol. 9, 3067. doi: 10.3389/fimmu.2018.03067 30619379 PMC6306490

[B98] Kim C. S. Ahmad S. Wu T. Walton W. G. Redinbo M. R. Tarran R. (2018). SPLUNC1 is an allosteric modulator of the epithelial sodium channel. FASEB J. 32 (5), 2478–2491. doi: 10.1096/fj.201701126R 29295861 PMC5901381

[B99] Klein J. O. (2000). The burden of otitis media. Vaccine 19, S2–S8. doi: 10.1016/S0264-410X(00)00271-1 11163456

[B100] Knowles M. R. Daniels L. A. Davis S. D. Zariwala M. A. Leigh M. W. (2013). Primary ciliary dyskinesia. recent advances in diagnostics, genetics, and characterization of clinical disease. Am. J. Respir. Crit. Care Med. 188 (8), 913–922. doi: 10.1164/rccm.201301-0059CI 23796196 PMC3826280

[B101] Kolpen M. Kragh K. N. Enciso J. B. Faurholt-Jepsen D. Lindegaard B. Egelund G. B.. (2022). Bacterial biofilms predominate in both acute and chronic human lung infections. Thorax. doi: 10.1136/thoraxjnl-2021-217576 PMC9510407 35017313

[B102] Koo H. Allan R. Howlin R. Stoodley P. Hall-Stoodley L. (2017). Targeting microbial biofilms: current and prospective therapeutic strategies. Nat. Rev. Microbiol. 15, 740–755. doi: 10.1038/nrmicro.2017.99 28944770 PMC5685531

[B103] Kragh K. N. Alhede M. Rybtke M. Stavnsberg C. Jensen PØ Tolker-Nielsen T.. (2018). The inoculation method could impact the outcome of microbiological experiments. Appl. Environ. Microbiol. 84 (5), e02264–e02217. doi: 10.1128/AEM.02264-17 29269495 PMC5812950

[B104] Kragh K. N. Hutchison J. B. Melaugh G. Rodesney C. Roberts A. E. Irie Y.. (2016). Role of multicellular aggregates in biofilm formation. mBio. 7 (2), e00237. doi: 10.1128/mBio.00237-16 27006463 PMC4807362

[B105] Hong K. U. Reynolds S. D. Watkins S. Fuchs E. Stripp B. R. Irie Y.. (2004). Basal cells are a multipotent progenitor capable of renewing the bronchial epithelium. Am J Pathol 164 (2), 577–88. doi: 10.1016/S0002-9440(10)63147-1 PMC1602270 14742263

[B106] Lai S. K. Wang Y. Y. Wirtz D. Hanes J. (2009). Micro- and macrorheology of mucus. Adv. Drug Delivery Rev. 61 (2), 86–100. doi: 10.1016/j.addr.2008.09.012 PMC2736374 19166889

[B107] Lam J. Chan R. Lam K. Costerton J. W. (1980). Production of mucoid microcolonies by pseudomonas aeruginosa within infected lungs in cystic fibrosis. Infect. Immun. 28, 546–556. doi: 10.1128/iai.28.2.546-556.1980 6772562 PMC550970

[B108] Leigh M. W. Pittman J. E. Carson J. L. Ferkol T. W. Dell S. D. Davis S. D.. (2009). Clinical and genetic aspects of primary ciliary dyskinesia/Kartagener syndrome. Genet. Med. 11 (7), 473–487. doi: 10.1097/GIM.0b013e3181a53562 19606528 PMC3739704

[B109] Levitsky (1982). “Pulmonary physiology,” in Respiratory physiology: the essentials (Baltimore, Md: Williams and Wilkins), 1–13. p. 32–48. McGraw-Hill, New York, N.Y., 53; West J. B. 1974.

[B110] Lichtenberg M. Line L. Schrameyer V. Jakobsen T. H. Rybtke M. L. Toyofuku M. (2021). Nitric-oxide-driven oxygen release in anoxic *Pseudomonas aeruginosa* . in iScience 24 (12), 103404. doi: 10.1016/j.isci.2021.103404 34849468 PMC8608891

[B111] Lieleg O. Caldara M. Baumgärtel R. Ribbeck K. (2011). Mechanical robustness of pseudomonas aeruginosa biofilms. Soft Matter. 7, 3307–3314. doi: 10.1039/c0sm01467b 21760831 PMC3134232

[B112] Limoli D. H. Jones C. J. Wozniak D. J. (2015). Bacterial extracellular polysaccharides in biofilm formation and function. Microbiol. Spectr. 3 (3), 10. doi: 10.1128/9781555817466.ch11 PMC4657554 26185074

[B113] Limoli D. H. Rockel A. B. Host K. M. Jha A. Kopp B. T. Hollis T.. (2014). Cationic antimicrobial peptides promote microbial mutagenesis and pathoadaptation in chronic infections. PloS Pathog. 10 (4), e1004083. doi: 10.1371/journal.ppat.1004083 24763694 PMC3999168

[B114] Liu Y. Di M. E. Chu H. W. Liu X. Wang L. Wenzel S.. (2013). Increased susceptibility to pulmonary pseudomonas infection in Splunc1 knockout mice. J. Immunol. 191 (8), 4259–4268. doi: 10.4049/jimmunol.1202340 24048904 PMC3839417

[B115] Lucas J. S. Barbato A. Collins S. A. Goutaki M. Behan L. Caudri D.. (2017). European Respiratory society guidelines for the diagnosis of primary ciliary dyskinesia. Eur. Respir. J. 49 (1), 1601090. doi: 10.1183/13993003.01090-2016 27836958 PMC6054534

[B116] Lucas J. S. Leigh M. W. (2014). Diagnosis of primary ciliary dyskinesia: searching for a gold standard. Eur. Respir. J. 44 (6), 1418–1422. doi: 10.1183/09031936.00175614 25435529

[B117] Luke N. R. Jurcisek J. A. Bakaletz L. O. Campagnari A. A. (2007). Contribution of moraxella catarrhalis type IV pili to nasopharyngeal colonization and biofilm formation. Infect. Immun. 75 (12), 5559–5564. doi: 10.1128/IAI.00946-07 17908808 PMC2168369

[B118] Mai-Prochnow A. Lucas-Elio P. Egan S. Thomas T Webb J. S. Sanchez-Amat A. (2008). Hydrogen peroxide linked to lysine oxidase activity facilitates biofilm differentiation and dispersal in several gram-negative bacteria. J Bacteriol. 190 (15), 5493–501. doi: 10.1128/JB.00549-08 18502869 PMC2493266

[B119] Malhotra S. Hayes D. Jr Wozniak D. J. (2019). Cystic fibrosis and pseudomonas aeruginosa: the host-microbe interface. Clin. Microbiol. Rev. 32 (3), e00138–e00118. doi: 10.1128/CMR.00138-18 31142499 PMC6589863

[B120] Malhotra S. Limoli D. H. English A. E. Parsek M. R. Wozniak D. J. (2018). Mixed communities of mucoid and nonmucoid pseudomonas aeruginosa exhibit enhanced resistance to host antimicrobials. mBio. 9 (2), e00275–e00218. doi: 10.1128/mBio.00275-18 29588399 PMC5874919

[B121] Man W. H. de Steenhuijsen Piters W. A. Bogaert D. (2017). The microbiota of the respiratory tract: gatekeeper to respiratory health. Nat. Rev. Microbiol. 15 (5), 259–270. doi: 10.1038/nrmicro.2017.14 28316330 PMC7097736

[B122] Matsui H. Grubb B. R. Tarran R. Randell S. H. Gatzy J. T. Davis C. W.. (1998). Evidence for periciliary liquid layer depletion, not abnormal ion composition, in the pathogenesis of cystic fibrosis airways disease. Cell 95 (7), 1005–1015. doi: 10.1016/S0092-8674(00)81724-9 9875854

[B123] Matsui H. Wagner V. E. Hill D. B. Schwab U. E. Rogers T. D. Button B.. (2006). A physical linkage between cystic fibrosis airway surface dehydration and pseudomonas aeruginosa biofilms. Proc. Natl. Acad. Sci. U.S.A. 103, 18131–18136. doi: 10.1073/pnas.0606428103 17116883 PMC1838718

[B124] Matsuyama M. Martins A. J. Shallom S. Kamenyeva O. Kashyap A. Sampaio E. P.. (2018). Transcriptional response of respiratory epithelium to nontuberculous mycobacteria. Am. J. Respir. Cell Mol. Biol. 58 (2), 241–252. doi: 10.1165/rcmb.2017-0218OC 28915071 PMC5806000

[B125] McGillivary G. Bakaletz L. O. (2010). The multifunctional host defense peptide SPLUNC1 is critical for homeostasis of the mammalian upper airway. PloS One 5 (10), e13224. doi: 10.1371/journal.pone.0013224 20949060 PMC2951362

[B126] Mishra M. Byrd M. S. Sergeant S. Azad A. K. Parsek M. R. McPhail L.. (2012). Pseudomonas aeruginosa psl polysaccharide reduces neutrophil phagocytosis and the oxidative response by limiting complement-mediated opsonization. Cell Microbiol. 14, 95–106. doi: 10.1111/j.1462-5822.2011.01704.x 21951860 PMC4466118

[B127] Miyamoto N. Bakaletz L. O. (1996). Selective adherence of non-typeable haemophilus influenzae (NTHi) to mucus or epithelial cells in the chinchilla eustachian tube and middle ear. Microb. Pathog. 21 (5), 343–356. doi: 10.1006/mpat.1996.0067 8938642

[B128] Mokrzan E. M. Dairo K. A. Novotny L. A. Bakaletz L. O. (2020). Nontypeable haemophilus influenzae responds to virus-infected cells with a significant increase in type IV pilus expression. mSphere 5, e00384-20. doi: 10.1128/mSphere.00384-20 32461275 PMC7253600

[B129] Møller M. E. Alanin M. C. Grønhøj C. Aanæs K. Høiby N. von Buchwald C. (2017). Sinus bacteriology in patients with cystic fibrosis or primary ciliary dyskinesia: A systematic review. Am. J. Rhinol. Allergy 31 (5), 293–298. doi: 10.2500/ajra.2017.31.4461 28859703 PMC5590177

[B130] Moreau-Marquis S. Bomberger J. M. Anderson G. G. Swiatecka-Urban A. Ye S. O’Toole G. A.. (2008). The DeltaF508-CFTR mutation results in increased biofilm formation by pseudomonas aeruginosa by increasing iron availability. Am. J. Physiol. Lung Cell. Mol. Physiol. 295, L25–L37. doi: 10.1152/ajplung.00391.2007 18359885 PMC2494796

[B131] Morris A. J. Jackson L. Cw Yau Y. Reichhardt C. Beaudoin T. Uwumarenogie S.. (2021). The role of psl in the failure to eradicate pseudomonas aeruginosa biofilms in children with cystic fibrosis. NPJ Biofilms Microbiomes 7 (1), 63. doi: 10.1038/s41522-021-00234-3 34349133 PMC8338932

[B132] Morrison C. B. Shaffer K. M. Araba K. C. Markovetz M. R. Wykoff J. A. Quinney N. L.. (2022). Treatment of cystic fibrosis airway cells with CFTR modulators reverses aberrant mucus properties *via* hydration. Eur. Respir. J. 59 (2), 2100185. doi: 10.1183/13993003.00185-2021 34172469 PMC8859811

[B133] Moser C. Jensen PØ Thomsen K. Kolpen M. Rybtke M. Lauland A. S.. (2021). Immune responses to pseudomonas aeruginosa biofilm infections. Front. Immunol. 12, 625597. doi: 10.3389/fimmu.2021.625597 33692800 PMC7937708

[B134] Nguyen D. Singh P. K. (2006). Evolving stealth: genetic adaptation of pseudomonas aeruginosa during cystic fibrosis infections. Proc. Natl. Acad. Sci. U.S.A. 103 (22), 8305–8306. doi: 10.1073/pnas.0602526103 16717189 PMC1482488

[B135] Nistico L. Kreft R. Gieseke A. Coticchia J. M. Burrows A. Khampang P.. (2011). Adenoid reservoir for pathogenic biofilm bacteria. J. Clin. Microbiol. 49 (4), 1411–1420. doi: 10.1128/JCM.00756-10 21307211 PMC3122793

[B136] Nolan L. M. Turnbull L. Katrib M. Osvath S. R. Losa D. Lazenby J. J.. (2020). Pseudomonas aeruginosa is capable of natural transformation in biofilms. Microbiology 166, 995–1003. doi: 10.1099/mic.0.000956 32749953 PMC7660920

[B137] Novotny L. A. Bakaletz L. O. (2016). Intercellular adhesion molecule 1 serves as a primary cognate receptor for the type IV pilus of nontypeable haemophilus influenzae. Cell. Microbiol. 18, 1043–1055. doi: 10.1111/cmi.12575 26857242 PMC4959963

[B138] Novotny L. A. Bakaletz L. O. (2020). Transcutaneous immunization with a nontypeable haemophilus influenzae dual adhesin-directed immunogen induces durable and boostable immunity. Vaccine 38 (10), 2378–2386. doi: 10.1016/j.vaccine.2020.01.052 32001071 PMC7219548

[B139] Novotny L. A. Mason K. M. Bakaletz L. O. (2005). Development of a chinchilla model to allow direct, continuous, biophotonic imaging of bioluminescent nontypeable haemophilus influenzae during experimental otitis media. Infect. Immun. 73 (1), 609–611. doi: 10.1128/IAI.73.1.609-611.2005 15618201 PMC538955

[B140] Overhage J. Campisano A. Bains M. Torfs E. C. Rehm B. H. Hancock R. E. (2008). Human host defense peptide LL-37 prevents bacterial biofilm formation. Infect. Immun. 76 (9), 4176–4182. doi: 10.1128/IAI.00318-08 18591225 PMC2519444

[B141] Parsek M. R. Singh P. K. (2003). Bacterial biofilms: an emerging link to disease pathogenesis. Annu. Rev. Microbiol. 57, 677–70114527295. doi: 10.1146/annurev.micro.57.030502.090720 14527295

[B142] Pestrak M. J. Chaney S. B. Eggleston H. C. Dellos-Nolan S. Dixit S. Mathew-Steiner S. S.. (2018). Pseudomonas aeruginosa rugose small-colony variants evade host clearance, are hyper-inflammatory, and persist in multiple host environments. PloS Pathog. 14 (2), e1006842. doi: 10.1371/journal.ppat.1006842 29394295 PMC5812653

[B143] Peterson B. W. He Y. Ren Y. Zerdoum A. Libera M. R. Sharma P. K.. (2015). Viscoelasticity of biofilms and their recalcitrance to mechanical and chemical challenges. FEMS Microbiol. Rev. 39 (2), 234–245. doi: 10.1093/femsre/fuu008 25725015 PMC4398279

[B144] Pezzulo A. A. Tang X. X. Hoegger M. J. Abou Alaiwa M. H. Ramachandran S. Moninger T. O.. (2012). Reduced airway surface pH impairs bacterial killing in the porcine cystic fibrosis lung. Nature 487 (7405), 109–113. doi: 10.1038/nature11130 22763554 PMC3390761

[B145] Pillarisetti N. Williamson E. Linnane B. Skoric B. Robertson C. F. Robinson P.. (2011). Infection, inflammation, and lung function decline in infants with cystic fibrosis. Am. J. Respir. Crit. Care Med. 184, 75. doi: 10.1164/rccm.201011-1892OC 21493738

[B146] Pittet L. .A. Hall-Stoodley L. Rutkowski M. R. Harmsen A. G.. (2010). Influenza virus infection decreases tracheal mucociliary velocity and clearance of Streptococcus pneumoniae. Am J Respir Cell Mol Biol. 42 (4), 450–60. doi: 10.1165/rcmb.2007-0417OC 19520922 PMC2848738

[B147] Post J. C. Stoodley P. Hall-Stoodley L. Ehrlich G. D. (2004). The role of biofilms in otolaryngologic infections. Curr. Opin. Otolaryngol Head Neck Surg. 12 (3), 185–190. doi: 10.1097/01.moo.0000124936.46948.6a 15167027

[B148] Prince A. A. Steiger J. D. Khalid A. N. Dogrhamji L. Reger C. Eau Claire S.. (2008). Prevalence of biofilm forming bacteria in chronic rhinosinusitis. Am. J. Rhinol. 22, 239–245. doi: 10.2500/ajr.2008.22.3180 18588755

[B149] Psaltis A. J. Ha K. R. Beule A. G. Tan L. W. Wormald P. J.. (2007). Confocal scanning laser microscopy evidence of biofilms in patients with chronic rhinosinusitis. Laryngoscope 117 (7), 1302–1306. doi: 10.1097/MLG.0b013e31806009b0 17603329

[B150] Røder H. L. Trivedi U. Russel J.. (2021). Biofilms can act as plasmid reserves in the absence of plasmid specific selection. NPJ Biofilms Microbiomes 7, 78. doi: 10.1038/s41522-021-00249-w 34620879 PMC8497521

[B151] Rayner R. E. Makena P. Prasad G. L. Cormet-Boyaka E. (2019). Optimization of normal human bronchial epithelial (NHBE) cell 3D cultures for *in vitro* lung model studies. Sci. Rep. 9 (1), 500. doi: 10.1038/s41598-018-36735-z 30679531 PMC6346027

[B152] Reynolds S. D. Zemke A. C. Giangreco A. Brockway B. L. Teisanu R. M. Drake J. A.. (2008). Conditional stabilization of beta-catenin expands the pool of lung stem cells. Stem Cells 26 (5), 1337–1346. doi: 10.1634/stemcells.2008-0053 18356571 PMC2773682

[B153] Rock J. R. Randell S. H. Hogan B. L. (2010). Airway basal stem cells: a perspective on their roles in epithelial homeostasis and remodeling. Dis. Model. Mech. 3 (9-10), 545–556. doi: 10.1242/dmm.006031 20699479 PMC2931533

[B154] Rogan M. P. Taggart C. C. Greene C. M. Murphy P. G. O'Neill S. J. McElvaney N. G. (2004). Loss of microbicidal activity and increased formation of biofilm due to decreased lactoferrin activity in patients with cystic fibrosis. J. Infect. Dis. 190 (7), 1245–1253. doi: 10.1086/423821 15346334

[B155] Römling U. (2019). Innate immune mechanisms with a focus on small-molecule microbe-host cross talk. J. Innate Immun. 11 (3), 191–192. doi: 10.1159/000495817 30726830 PMC6738236

[B156] Samuels T. L. Yan J. C. Khampang P. Dettmar P. W. MacKinnon A. Hong W.. (2017). Association of Gel-Forming Mucins and Aquaporin Gene Expression With Hearing Loss, Effusion Viscosity, and Inflammation in Otitis Media With Effusion. JAMA Otolaryngol Head Neck Surg 149 (8), 810–817. doi: 10.1001/jamaoto.2017.0386 PMC5710562 28594978

[B157] Sánchez Montalvo A. Gohy S. Rombaux P. Pilette C and Hox V. (2022). The role of IgA in chronic upper airway disease: Friend or foe? Front. Allergy 3, 852546. doi: 10.3389/falgy.2022.852546 35386640 PMC8974816

[B158] Sanclement J. A. Webster P. Thomas J. Ramadan H. H. (2005). Bacterial biofilms in surgical specimens of patients with chronic rhinosinusitis. Laryngoscope 115 (4), 578–582. doi: 10.1097/01.mlg.0000161346.30752.18 15805862

[B159] Sanderson A. R. Leid J. G. Hunsaker D. (2006). Bacterial biofilms on the sinus mucosa of human subjects with chronic rhinosinusitis. Laryngoscope 116 (7), 1121–1126. doi: 10.1097/01.mlg.0000221954.05467.54 16826045

[B160] Schobert M. Jahn D. (2010). Anaerobic physiology of pseudomonas aeruginosa in the cystic fibrosis lung. Int. J. Med. Microbiol. 300 (8), 549–556. doi: 10.1016/j.ijmm.2010.08.007 20951638

[B161] Secor P. R. Michaels L. A. Ratjen A. Jennings L. K. Singh P. K. (2018). Entropically driven aggregation of bacteria by host polymers promotes antibiotic tolerance in pseudomonas aeruginosa. Proc. Natl. Acad. Sci. U.S.A. 115 (42), 10780–10785. doi: 10.1073/pnas.1806005115 30275316 PMC6196481

[B162] Sethi S. Murphy T. F. (2008). Infection in the pathogenesis and course of chronic obstructive pulmonary disease. N Engl. J. Med. 359 (22), 2355–2365. doi: 10.1056/NEJMra0800353 19038881

[B163] Shah V. S. Meyerholz D. K. Tang X. X. Reznikov L. Abou Alaiwa M. Ernst S. E.. (2016). Airway acidification initiates host defense abnormalities in cystic fibrosis mice. Science 351 (6272), 503–507. doi: 10.1126/science.aad5589 26823428 PMC4852973

[B164] Shapiro A. J. Zariwala M. A. Ferkol T. Davis S. D. Sagel S. D. Dell S. D.. (2016). Diagnosis, monitoring, and treatment of primary ciliary dyskinesia: PCD foundation consensus recommendations based on state-of-the art review. Pediatr. Pulmonol. 51 (2), 115–132. doi: 10.1002/ppul.23304 26418604 PMC4912005

[B165] Sibila O. Perea L. Cantó E. Shoemark A. Cassidy D. Smith A. H.. (2019). Antimicrobial peptides, disease severity and exacerbations in bronchiectasis. Thorax 74 (9), 835–842. doi: 10.1136/thoraxjnl-2018-212895 31278172

[B166] Singhal D. Foreman A. Jervis-Bardy J. Wormald P. J. (2011). Staphylococcus aureus biofilms: nemesis of endoscopic sinus surgery. Laryngoscope 121, 1578–1583. doi: 10.1002/lary.21805 21647904

[B167] Singhal D. Psaltis A. J. Foreman A. Wormald P. J. (2010). The impact of biofilms on outcomes after endoscopic sinus surgery. Am. J. Rhinol. Allergy 24 (3), 169–174. doi: 10.2500/ajra.2010.24.3462 20537281

[B168] Singh P. K. Parsek M. R. Greenberg E. P. Welsh M. J. (2002). A component of innate immunity prevents bacterial biofilm development. Nature 417 (6888), 552–555. doi: 10.1038/417552a 12037568

[B169] Singh P. K. Schaefer A. L. Parsek M. R. Moninger T. O. Welsh M. J. Greenberg E. P. (2000). Quorum-sensing signals indicate that cystic fibrosis lungs are infected with bacterial biofilms. Nature 407 (6805), 762–764. doi: 10.1038/35037627 11048725

[B170] Smith E. E. Buckley D. G. Wu Z. Saenphimmachak C. Hoffman L. R. D'Argenio D. A.. (2006). Genetic adaptation by pseudomonas aeruginosa to the airways of cystic fibrosis patients. Proc. Natl. Acad. Sci. U.S.A. 103 (22), 8487–8492. doi: 10.1073/pnas.0602138103 16687478 PMC1482519

[B171] Sommer L. M. Alanin M. C. Marvig R. L. Nielsen K. G. Høiby N. von Buchwald C.. (2016). Bacterial evolution in PCD and CF patients follows the same mutational steps. Sci. Rep. 6, 28732. doi: 10.1038/srep28732 27349973 PMC4923847

[B172] Starner T. D. Zhang N. Kim G. Apicella M. A. McCray P. B. Jr. (2006). Haemophilus influenzae forms biofilms on airway epithelia: implications in cystic fibrosis. Am. J. Respir. Crit. Care Med. 174 (2), 213–220. doi: 10.1164/rccm.200509-1459OC 16675778 PMC2662906

[B173] Staudinger B. J. Muller J. F. Halldórsson S. Boles B. Angermeyer A. Nguyen D.. (2014). Conditions associated with the cystic fibrosis defect promote chronic pseudomonas aeruginosa infection. Am. J. Respir. Crit. Care Med. 189 (7), 812–824. doi: 10.1164/rccm.201312-2142OC 24467627 PMC4225830

[B174] Stewart P. S. (1996). Theoretical aspects of antibiotic diffusion into microbial biofilms. Antimicrob. Agents Chemother. 40 (11), 2517–2522. doi: 10.1128/AAC.40.11.2517 8913456 PMC163567

[B175] Stewart P. S. (2015). Antimicrobial tolerance in biofilms. Microbiol. Spectr. 3 (3), 10. doi: 10.1128/9781555817466.ch13 PMC4507308 26185072

[B176] Tecle T. Tripathi S. Hartshorn K. L. (2010). Defensins and cathelicidins in lung immunity. Innate Immun. 16 (3), 151–159. doi: 10.1177/1753425910365734 20418263

[B177] Thomsen K. Kobayashi O. Kishi K. Shirai R. Østrup Jensen P. Heydorn A.. (2022). Animal models of chronic and recurrent pseudomonas aeruginosa lung infection: significance of macrolide treatment. APMIS 130 (7), 458–476. doi: 10.1111/apm.13161 34117660

[B178] Thornton R. B. Rigby P. J. Wiertsema S. P. Filion P. Langlands J. Coates H. L.. (2011). Multi-species bacterial biofilm and intracellular infection in otitis media. BMC Pediatr. 11, 94. doi: 10.1186/1471-2431-11-94 22018357 PMC3224757

[B179] Thornton R. B. Wiertsema S. P. Kirkham L. A. Rigby P. J. Vijayasekaran S. Coates H. L.. (2013). Neutrophil extracellular traps and bacterial biofilms in middle ear effusion of children with recurrent acute otitis media-a potential treatment target. PloS One 8 (2), e53837. doi: 10.1371/journal.pone.0053837 23393551 PMC3564866

[B180] Toone S. L. Ratkiewicz M. Novotny L. A. Phong B. L. Bakaletz L. O. (2020). Nontypeable haemophilus influenzae type IV pilus mediates augmented adherence to rhinovirus-infected human airway epithelial cells. Infect. Immun. 88, e00248–e00220. doi: 10.1128/IAI.00248-20 32540869 PMC7440766

[B181] Ubell M. L. Kerschner J. E. Wackym P. A. Burrows A. (2008). MUC2 expression in human middle ear epithelium of patients with otitis media. Arch. Otolaryngol Head Neck Surg. 134 (1), 39–44. doi: 10.1001/archoto.2007.10 18209134 PMC2912163

[B182] Wagner C. E. Wheeler K. M. Ribbeck K. (2018). Mucins and their role in shaping the functions of mucus barriers. Annu. Rev. Cell Dev. Biol. 34, 189–215. doi: 10.1146/annurev-cellbio-100617-062818 30296390 PMC11906035

[B183] Walker T. S. Tomlin K. L. Worthen G. S. Poch K. R. Lieber J. G. Saavedra M. T. (2018). Enhanced Pseudomonas aeruginosa biofilm development mediated by human neutrophils. Infect Immun. 73 (6), 3696–701. doi: 10.1128/IAI.73.6.3693-3701.2005 PMC1111839 15908399

[B184] Walker W. T. Jackson C. L. Allan R. N. Collins S. A. Kelso M. J. Rineh A.. (2017). Primary ciliary dyskinesia ciliated airway cells show increased susceptibility to haemophilus influenzae biofilm formation. Eur. Respir. J. 50 (3), 1700612. doi: 10.1183/13993003.00612-2017 28890436

[B185] Walker W. T. Jackson C. L. Coles J. Lackie P. M. Faust S. N. Hall-Stoodley L.. (2014). Ciliated cultures from patients with primary ciliary dyskinesia produce nitric oxide in response to haemophilus influenzae infection and proinflammatory cytokines. Chest 145 (3), 668–669. doi: 10.1378/chest.13-2398 24590042

[B186] Walker W. T. Jackson C. L. Lackie P. M. Hogg C. Lucas J. S. (2012). Nitric oxide in primary ciliary dyskinesia. Eur. Respir. J. 40 (4), 1024–1032. doi: 10.1183/09031936.00176111 22408195

[B187] Walters M. C. Roe F. Bugnicourt A. Franklin M. J. Stewart P. S. (2003). Contributions of antibiotic penetration, oxygen limitation, and low metabolic activity to tolerance of pseudomonas aeruginosa biofilms to ciprofloxacin and tobramycin. Antimicrob. Agents Chemother. 47, 317–323. doi: 10.1128/AAC.47.1.317-323.2003 12499208 PMC148957

[B188] Welp A. L. Bomberger J. M. (2020). Bacterial community interactions during chronic respiratory disease. Front. Cell Infect. Microbiol. 10, 213. doi: 10.3389/fcimb.2020.00213 32477966 PMC7240048

[B189] Welsh M. J. Rogers C. S. Stoltz D. A. Meyerholz D. K. Prather R. S. (2009). Development of a porcine model of cystic fibrosis. Trans. Am. Clin. Climatol Assoc. 120, 149–162. 19768173 PMC2744522

[B190] Wheeler K. M. Cárcamo-Oyarce G. Turner B. S. Dellos-Nolan S. Co J. Y. Lehoux S.. (2019). Mucin glycans attenuate the virulence of pseudomonas aeruginosa in infection. Nat. Microbiol. 4 (12), 2146–2154. doi: 10.1038/s41564-019-0581-8 31611643 PMC7157942

[B191] Whitchurch C. B. Tolker-Nielsen T. Ragas P. C. Mattick J. S.. (2002). Extracellular DNA required for bacterial biofilm formation. Science 295 (559), 1487. doi: 10.1126/science.295.5559.1487 11859186

[B192] Whitsett J. A. (2018). Airway epithelial differentiation and mucociliary clearance. Ann. Am. Thorac. Soc 15 (Suppl 3), S143–S148. doi: 10.1513/AnnalsATS.201802-128AW 30431340 PMC6322033

[B193] Whitsett J. A. Alenghat T. (2015). Respiratory epithelial cells orchestrate pulmonary innate immunity. Nat. Immunol. 16, 27–35. doi: 10.1038/ni.3045 25521682 PMC4318521

[B194] Worlitzsch D. Tarran R. Ulrich M. Schwab U. Cekici A. Meyer K. C.. (2002). Effects of reduced mucus oxygen concentration in airway pseudomonas infections of cystic fibrosis patients. J. Clin. Invest. 109 (3), 317–325. doi: 10.1172/JCI0213870 11827991 PMC150856

[B195] Zayas J. G. Man G. C. King M. (1990). Tracheal mucus rheology in patients undergoing diagnostic bronchoscopy. Am. Rev. Respir. Dis. 141, 1107–1113. doi: 10.1164/ajrccm/141.5_Pt_1.1107 2339832

[B196] Zemke A. C. D'Amico E. J. Snell E. C. Torres A. M. Kasturiarachi N. Bomberger J. M. (2020). Dispersal of epithelium-associated pseudomonas aeruginosa biofilms. mSphere. 5 (4), e00630–e00620. doi: 10.1128/mSphere.00630-20 32669459 PMC7364222

